# Effectiveness of a multifactorial intervention for dizziness in older people in primary care: A cluster randomised controlled trial

**DOI:** 10.1371/journal.pone.0204876

**Published:** 2018-10-09

**Authors:** Hanneke Stam, Johannes C. van der Wouden, Jacqueline G. Hugtenburg, Jos W. R. Twisk, Henriëtte E. van der Horst, Otto R. Maarsingh

**Affiliations:** 1 Department of General Practice and Elderly Care Medicine, Amsterdam Public Health Research Institute, Amsterdam UMC, Vrije Universiteit, Amsterdam, The Netherlands; 2 Department of Clinical Pharmacology and Pharmacy, Amsterdam UMC, Vrije Universiteit, Amsterdam, The Netherlands; 3 Department of Clinical Epidemiology and Biostatistics, Amsterdam UMC, Vrije Universiteit, Amsterdam, The Netherlands; University of Glasgow, UNITED KINGDOM

## Abstract

**Objectives:**

Dizziness is common in older people. Physicians are often unable to identify a specific cause for dizziness in older people, even after an extensive diagnostic work-up. A prognosis-oriented approach, i.e. treating modifiable risk factors for an unfavourable course of dizziness, may reduce dizziness-related impairment in older people in primary care.

**Design:**

Cluster randomized controlled trial.

**Setting:**

45 primary care practices in The Netherlands.

**Participants:**

168 participants aged ≥65y who consulted their general practitioner for dizziness and experienced significant dizziness-related impairment (Dizziness Handicap Inventory (DHI) ≥30). Participants were part of to the intervention group (n = 83) or control group (n = 85), depending on whether they were enlisted in an intervention practice or in a control practice.

**Interventions:**

The multifactorial intervention consisted of: medication adjustment in case of ≥3 prescribed fall-risk-increasing drugs (FRIDs) and/or stepped mental health care in case of anxiety disorder and/or depression and/or exercise therapy in case of impaired functional mobility. The intervention was compared to usual care.

**Outcome measures:**

The primary outcome was dizziness-related impairment. Secondary outcomes were quality of life (QoL), dizziness frequency, fall frequency, anxiety and depression, use of FRIDs.

**Results:**

Intention-to-treat analysis showed no significant intervention effect on dizziness-related impairment (DHI score difference -0.69 [95% CI -5.66;4.28]; p = 0.79). The intervention proved effective in reducing the number of FRIDs (FRID difference -0.48 [95% CI -0.89;-0.06]; p = 0.02). No significant intervention effects were found on other secondary outcomes. The uptake of and adherence to the interventions was significantly lower in patients eligible for ≥2 interventions compared to patients eligible for one intervention (p<0.001).

**Conclusions:**

The multifactorial intervention for dizziness in older patients showed no significant intervention effect on most outcomes and adherence to the multifactorial intervention was low. Although multifactorial treatment for older dizzy people seems promising in theory, we question its feasibility in daily practice. Future research could focus on a sequential treatment for dizziness, e.g. measuring effectiveness of various evidence-based therapies in a stepwise approach.

## Introduction

Dizziness is a common health problem in older people. The prevalence of dizziness in people above 65 years of age ranges from 8% in the primary care population to 30% in the community. [1–7]. Dizziness strongly affects daily functioning in older adults [8–11], and is associated with depression, a lower self-rated health, and reduced social activity [8,12]. Older people with dizziness also have an increased risk of falling [13]. Most guidelines on dizziness promote a diagnosis-oriented approach, starting with a search for its cause followed by treatment once the underlying illness has been diagnosed [14,15]. If an accurate diagnosis of dizziness has been established, there is potential for effective treatment, such as the Epley manoeuvre for benign paroxysmal positional vertigo (BPPV) [16,17], or vestibular rehabilitation for persisting vertigo after the Epley manoeuvre or for chronic vertigo symptoms in patients who suffer from vestibular neuronitis or Méniѐre’s disease [18]. However, in older people, dizziness is often a diagnostic challenge because it can refer to a variety of sensations and there are many potential causes. Dizziness in the aged is likely to constitute a geriatric syndrome, i.e. caused by multiple contributing factors, involving several organ systems [7,19–21]. In up to 40% of older patients with dizziness physicians have difficulties in establishing a diagnosis, which might be due to the potential multifactorial origin and the broad etiologic spectrum of dizziness [1,2]. Moreover, if an accurate diagnosis of dizziness has been established, appropriate treatment may be lacking as is sometimes the case with polyneuropathy or orthostatic hypotension. Considering the high rate of older patients with unknown cause of dizziness or inability to treat its cause, a prognosis-oriented approach might add to the diagnosis-oriented approach [22,23]. A prognosis-oriented approach implies that after estimating the prognosis in a specific patient, potentially modifiable risk factors for an unfavourable outcome are targeted. By doing so, older patients with dizziness can be treated without knowing the precise cause of dizziness.

To evaluate the effectiveness of a prognosis-oriented approach in older people with dizziness in primary care we compared a multifactorial risk factor guided intervention with usual care. The aim of the intervention was to target three modifiable risk factors for an unfavourable outcome of dizziness [24], and the intervention consisted of (1) medication adjustment in case of ≥3 fall-risk-increasing drugs (FRIDs), (2) stepped mental health care in case of anxiety disorder and/or depression, and (3) exercise therapy in case of impaired functional mobility. The multifactorial risk factor guided intervention aimed to reduce dizziness-related impairment.

## Materials and methods

The Reduction Of Dizziness in older pEOple (RODEO) study is a cluster randomised trial, assessing the effectiveness of a multifactorial risk factor guided intervention for dizziness in primary care. A detailed description of the study protocol has been published elsewhere [25]. Patients meeting the following inclusion criteria were eligible for enrolment: aged ≥65 years, having consulted their general practitioner (GP) for dizziness in the preceding 3 months and experiencing significant dizziness-related impairment (Dizziness Handicap Inventory (DHI) score ≥30) [26–28]. Dizziness was defined as a giddy or rotational sensation, loss of balance, faint feeling, light-headedness, instability, and/or tendency to fall. Patients with severe cognitive impairment, terminal illness, severe psychiatric problems, and insufficient mastery of Dutch were excluded. Patients were recruited from 45 primary care practices in the Netherlands between January 2015 and July 2016. All patients were visited at home for baseline assessment. Before the start of baseline assessment, written informed consent was obtained from each patient.

The study was registered at the Netherlands Trial Register (NTR4346). The study was approved by the Medical Ethics Review Committee of VU University Medical Center Amsterdam (approval number: NL49604.029.14), and was conducted according to the principles of the Declaration of Helsinki (version 2013) and the Dutch Medical Research Involving Human Subjects Act (WMO). For this paper, we followed the Consolidated Standards of Reporting Trials (CONSORT) statement with extension to cluster randomised trials [29].

### Intervention

All patients of the intervention group received one, two, or three risk factor guided interventions. The offered interventions were: (1) FRID medication adjustment in case of ≥3 prescribed FRIDs; (2) stepped mental health care in case of anxiety disorder and/or depression; and (3) exercise therapy in case of impaired functional mobility. When more than one intervention was applicable, these were started simultaneously. All intervention patients were contacted by phone to inform them about the intervention(s) that were suitable for them. When patients hesitated to start one or more interventions we gave them extra information by phone and in case of physical barriers to start one or more interventions we tried to tailor the intervention to the abilities of the patient. For example, if a patient was unable to visit the GP practice or physiotherapist, we arranged that the interventions took place at the patient’s home.

Patients in the intervention group had unrestricted access to usual care: no treatment was postponed or denied to participants. Blinding of patients and health care professionals was not possible due to the nature of the interventions.

#### FRID medication adjustment

The list of FRIDs included psychotropic drugs (sedatives, antidepressants, and neuroleptics), cardiovascular drugs (antihypertensives, nitrates, anti-arrhythmics, nicotinic acid, and β-adrenoceptor blocker eye drops), and other drugs (analgesics, anti-vertiginous drugs, hypoglycaemics, and urinary antispasmodics) [30]. A pharmacist and an independent GP or elderly care physician reviewed the FRID use for all patients with ≥3 prescribed FRIDs. This resulted in an individual FRID medication advice for each patient. If stopping entailed no health risks, the participant received the advice to stop the FRID(s). If stopping was not an option, the advice was to lower the dose or switch to an alternative drug. Patients were invited for a consultation with their own GP to discuss the FRID medication advice. FRID medication adjustment only took place if both the GP and the patient agreed.

#### Stepped mental health care

Patients were offered this intervention if they had a generalized anxiety disorder, panic disorder and/or major depressive disorder, assessed with the Generalized Anxiety Disorder-7 questionnaire (GAD-7) [31], Patient Health Questionnaire Panic Module (PHQ-PD) [32] and Patient Health Questionnaire-9 (PHQ-9) [33], respectively. The offered stepped care program involved four subsequent treatment steps, lasting 6 weeks each: watchful waiting (step 1), guided self-help treatment (step 2), Problem Solving Treatment (step 3) and referral to the GP to assess the appropriate next therapy for the patient (step 4). A mental health nurse practitioner, working in the patient’s own general practice, guided the patient through the program. Patient flow through the stepped care program depended on the patient’s symptoms level.

#### Exercise therapy

The presence of impaired functional mobility was defined as a Timed Up-and-Go (TUG) score of 20 seconds or more [34]. Patients with impaired functional mobility received standardized exercise therapy by a physiotherapist, one hour twice a week for eight weeks. The aim of exercise therapy was to improve strength and balance. A treatment protocol for the physiotherapists prescribed what exercises should be carried out every week and included pictures of the specific exercises.

### Usual care

Patients in the control group had unrestricted access to usual care: no treatment was postponed or denied to participants. GPs of control practices were not informed about the intervention and did not receive any training. Instead, they were asked to provide care as recommended in the guideline “Dizziness” of the Dutch College of General Practitioners (see S1 File) [14].

### Outcome measures

Patients were assessed at baseline and after three, six and 12 months. The primary outcome measure was dizziness-related impairment, assessed with the DHI. The DHI is a widely used self-report questionnaire, designed to quantify the impact of dizziness on everyday life. The difference in 1-year DHI score change between the intervention and control group was analysed. Secondary outcomes included quality of life (QoL; utility score (Dutch Tariff) [35] and visual analogue scale (VAS) score of EQ-5D-5L questionnaire) [36]; dizziness frequency and fall frequency (weekly assessed with a calendar); number of FRIDs; and presence of anxiety disorder and depression (assessed by using GAD-7, PHQ-PD, and PHQ-9) [31–33]. Except for number of FRIDs, all outcome measures were self-reported with the participants being the outcome assessors. Blinding of outcome assessors was therefore not possible. Although we also defined health care utilisation as secondary outcome in our study protocol, we did not succeed in assessing health care utilisation. We planned to extract health care utilisation data from the electronic medical records of the 45 primary care practices, but decided not to collect these because of quality concerns.

### Sample size and randomisation

The sample size calculation was based on a clinically relevant DHI score change of ≥11 points between the intervention group and control group and a standard deviation of 12.64 [28]. With α 0.05, β 0.20, an estimated intraclass correlation coefficient of 0.05 for clustering within practices, and the assumption of a loss to follow-up of 20%, 200 patients (100 in each group) were required. Cluster randomisation at practice level was conducted to avoid contamination. Practices were randomised by a researcher who was blinded to their identity (concealment of allocation) before the inclusion of patients began. Practices were stratified by list size into three strata: practices with up to 400, 400 to 800, and over 800 patients of 65 years and older. For each stratum, block randomisation with varying block size was used to create similar distributions in both study arms. The investigator was blinded to the size of each block.

### Statistical analysis

We used descriptive statistics for baseline characteristics and compared uptake of and adherence to the separate components of the intervention using chi-square tests. We conducted an intention-to-treat analysis applying linear mixed model analysis (continuous outcome variables), generalized estimating equation analysis (binary outcome variables) and negative binomial mixed model analysis (count outcome variable). All these analyses were adjusted for correlation between repeated measures within the same patient and are capable of handling missing data [37]. Because in mixed model analysis adjustment for more than two levels is possible, we investigated the effect of adjusting for general practice because of potential correlation between measurements of patients within the same practice. However, adding practices as a third level to the mixed model analyses did not significantly improve the models and did not change the results. Therefore, general practice was not included as an extra level in the final analyses. For all outcome measures, respondents were included in the analysis if at least one follow-up measurement was available. For all analyses, an adjustment was made for the baseline value of the particular outcome variable and the overall intervention effect over time and the intervention effect at three, six and 12 months follow-up were evaluated. For the intervention effect at three, six and 12 months, time and the interaction between intervention and time were added to the models. For dizziness frequency and fall frequency, both measured weekly during one year, the overall intervention effect over time was investigated. For number of FRIDs, which was measured at baseline and at 12 months, only the overall intervention effect could be analysed. We performed a crude analysis and an adjusted analysis for every outcome measure. The adjusted model included age, sex, living alone or not, polypharmacy, dizziness onset, dizziness-related impairment (DHI score), number of chronic diseases, psychiatric disorder, cardiovascular disease and functional mobility (TUG score).

We repeated all analyses applying the per-protocol principle for the intervention group. Intervention patients were included in the per protocol analyses if they fulfilled adherence criteria for at least one of three interventions. Adherence criteria for the interventions were assessed as follows: 1) FRID medication review resulting in at least one adopted medication advice; 2) finished stepped mental health care because of a reduction of anxiety and/or depressive symptoms or getting through to step four of the program; 3) at least eight training sessions of exercise therapy. All patients of the control group were included in the per-protocol analysis.

Data were analysed using IBM SPSS Statistics version 22 and Stata version 14.

## Results

### Recruitment and baseline characteristics of study population

**Fig 1:**
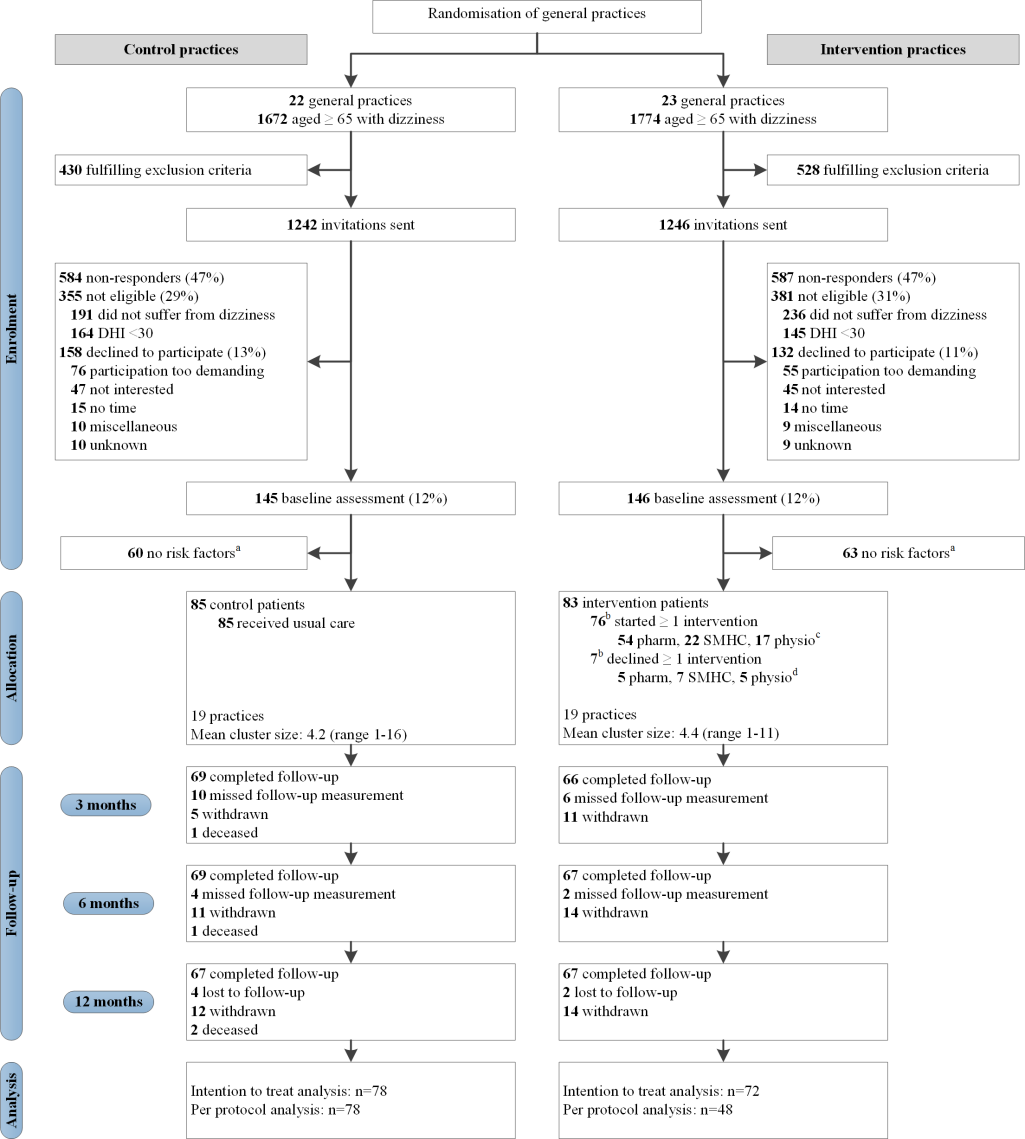
Flow of study participants. Abbreviations: *DHI* Dizziness Handicap Inventory; *pharm* fall risk increasing drug medication adjustment; *SMHC* stepped mental health care; *physio* exercise therapy. ^a^ at risk: usage of ≥ 3 Fall-risk-increasing drugs and/or presence of depressive or anxiety disorder and/or impaired functional mobility. ^b^ does not add up to 83: 7 patients did not start any intervention, 8 patients refused 1 intervention but started 1 or 2 other interventions. ^c^ does not add up to 76: 61 patients started 1 intervention, 13 patients started 2 interventions, 2 patients started 3 interventions. ^d^ does not add up to 17: 5 patients declined 1 intervention, 2 patients declined 2 interventions, 8 patients declined 1 intervention but started ≥1 other interventions.

Fig 1 gives an overview of enrolment, allocation and follow-up of study participants. A total of 168 patients were included: 83 patients in the intervention group and 85 patients in the control group. Table 1 summarizes the participants’ demographic and clinical characteristics. The mean age of the population was 78.8 years (standard deviation (SD) 7.3) and the majority of the patients were female (68.5%). Participants had a mean of 2.5 (SD 1.4) chronic diseases and 7.4 (SD 3.5) medication prescriptions. The mean DHI score at baseline was 51.0 (SD 15.1).

**Table 1:** Patient characteristics measured at baseline.*.

		Intervention group (n = 83)	Control group (n = 85)
**Demographic characteristics**			
Women		58 (69.9)	57 (67.1)
Age (years), mean ±SD (range 65–96)		78.6 ±7.0	79.0 ±7.6
Ethnicity			
	Dutch native	67 (80.7)	63 (74.1)
	Western immigrant	6 (7.2)	15 (17.6)
	Non-Western immigrant	10 (12.0)	7 (8.2)
Level of education			
	Elementary school	14 (16.9)	9 (10.6)
	High school	49 (59.0)	58 (68.2)
	College/university	20 (24.1)	18 (21.2)
Living situation			
	Alone	64 (77.1)	53 (62.4)
	Together	19 (22.9)	32 (37.6)
**Health characteristics**			
Cardiovascular disease		72 (86.7)	74 (87.1)
Neurological disease		34 (41.0)	28 (32.9)
Psychiatric disease^a^		28 (34.1)	30 (35.3)
	Presence of depressive disorder	11 (13.4)	7 (8.2)
	Presence of anxiety disorder	24 (29.3)	23 (27.1)
	Presence of panic disorder	4 (4.9)	8 (9.4)
Diabetes mellitus		21 (25.3)	22 (25.9)
Number of chronic diseases, mean ±SD (range 0–6)		2.4 ±1.4	2.5 ±1.4
Impaired functional mobility		21 (25.3)	25 (29.4)
Falling ≥1 time last year		43 (51.8)	46 (54.1)
EQ-5D-5L utility, mean ±SD (range -0.34–1.00)		0.0.59 ±0.0.26	0.66 ±0.22
EQ-5D-5L VAS, mean ±SD (range 20–95)		63.8 ±14.7	64.8 ±16.2
**Dizziness characteristics**			
DHI score, mean ±SD (range 30–88)		53.8 ±15.4	48.2 ±14.4
Onset of dizziness			
	1–4 weeks	1 (1.2)	2 (2.4)
	1–6 months	11 (13.3)	12 (14.1)
	6–48 months	15 (18.1)	25 (29.4)
	2–10 years	42 (50.6)	31 (36.5)
	> 10 years	14 (16.9)	15 (17.6)
Description of dizziness^b^			
	Instability or unsteadiness	68 (81.9)	65 (76.5)
	Loss of balance	70 (84.3)	68 (80.0)
	Light-headedness	71 (85.5)	58 (68.2)
	Rotational sensation	63 (75.9)	58 (68.2)
	Tendency to fall	56 (67.5)	58 (68.2)
	Giddy	45 (54.2)	48 (56.5)
	Environment spinning	30 (36.1)	40 (47.1)
	Becoming unwell	31 (37.3)	26 (30.6)
	Near faint	20 (24.1)	23 (27.1)
	Everything turning black	17 (20.5)	23 (27.1)
**Medication characteristics**			
No of drugs, mean ±SD (range 0–17)		7.2 ±3.5	7.6 ±3.4
Polypharmacy		61 (73.5)	73 (85.9)
Number of FRIDs, mean ±SD (range 0–10)		3.2 ±1.8	3.5 ±2.0
≥ 3 FRIDs		59 (71.1)	62 (72.9)

* Figures are numbers (percentages) unless stated otherwise.

Abbreviations: SD standard deviation; EQ-5D-5L Euro Quality of Life–5-dimension, 5-level questionnaire

IQR inter quartile range; VAS visual analogue scale; FRIDs Fall-risk-increasing drugs

^a^ Some patients had more than one psychiatric disorder

^b^ Adds up to more than 100%, because more than 1 answer was possible

### Uptake of the interventions

**Fig 2:**
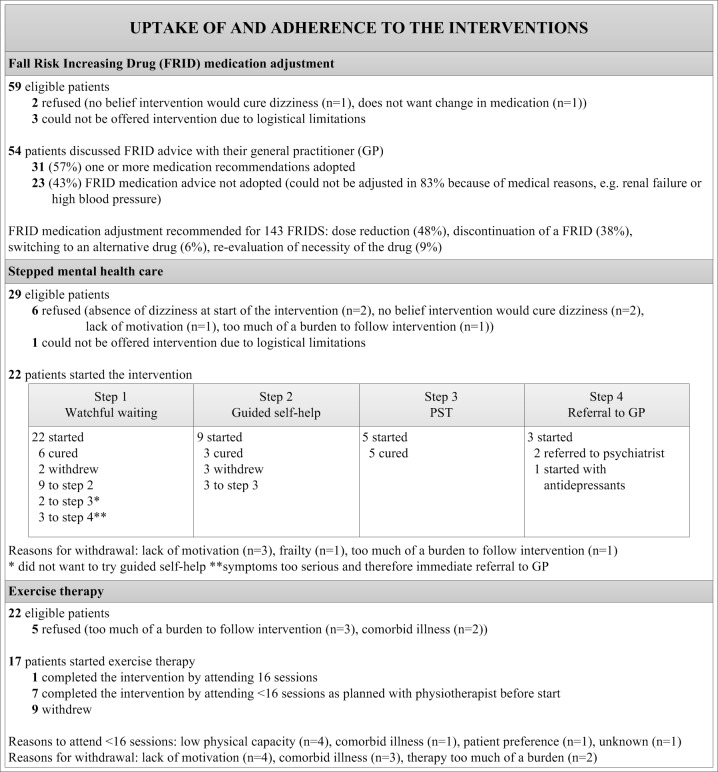
Uptake of and adherence to the interventions.

Of the 83 patients in the intervention group, 59 patients were eligible for FRID medication adjustment, 29 for stepped mental health care and 22 for exercise therapy. Fig 2 gives an overview of the uptake of and adherence to the interventions separately. Refusal and withdrawal were significantly higher for stepped mental health care and exercise therapy, as compared to FRID adjustment (p <0.001). Of the 60 patients eligible for a single intervention, 50 completed the intervention, five withdrew and five refused the intervention. Of the 19 patients eligible for two interventions, five completed both interventions, 13 refused (n = 8) or withdrew from (n = 5) at least one intervention. Of the four patients eligible for three interventions, one completed these interventions, three refused (n = 2) or withdrew from (n = 1) at least one intervention. Refusal and withdrawal were significantly higher in patients eligible for two or three interventions compared to patients eligible for one intervention (*p* <0.001).

### Numbers analysed

Data on the primary outcome on at least one follow-up moment were available from 72/83 (86.7%) patients in the intervention group and 78/85 (91.8%) patients in the control group, which was not statistically different [χ^2^(1) = 1.105, p = 0.293]. Per protocol analysis of the primary outcome was conducted with data of 48 patients of the intervention group and all patients of the control group (n = 72). Fig 1 provides more detail about follow-up assessments.

### Primary outcome

**Fig 3:**
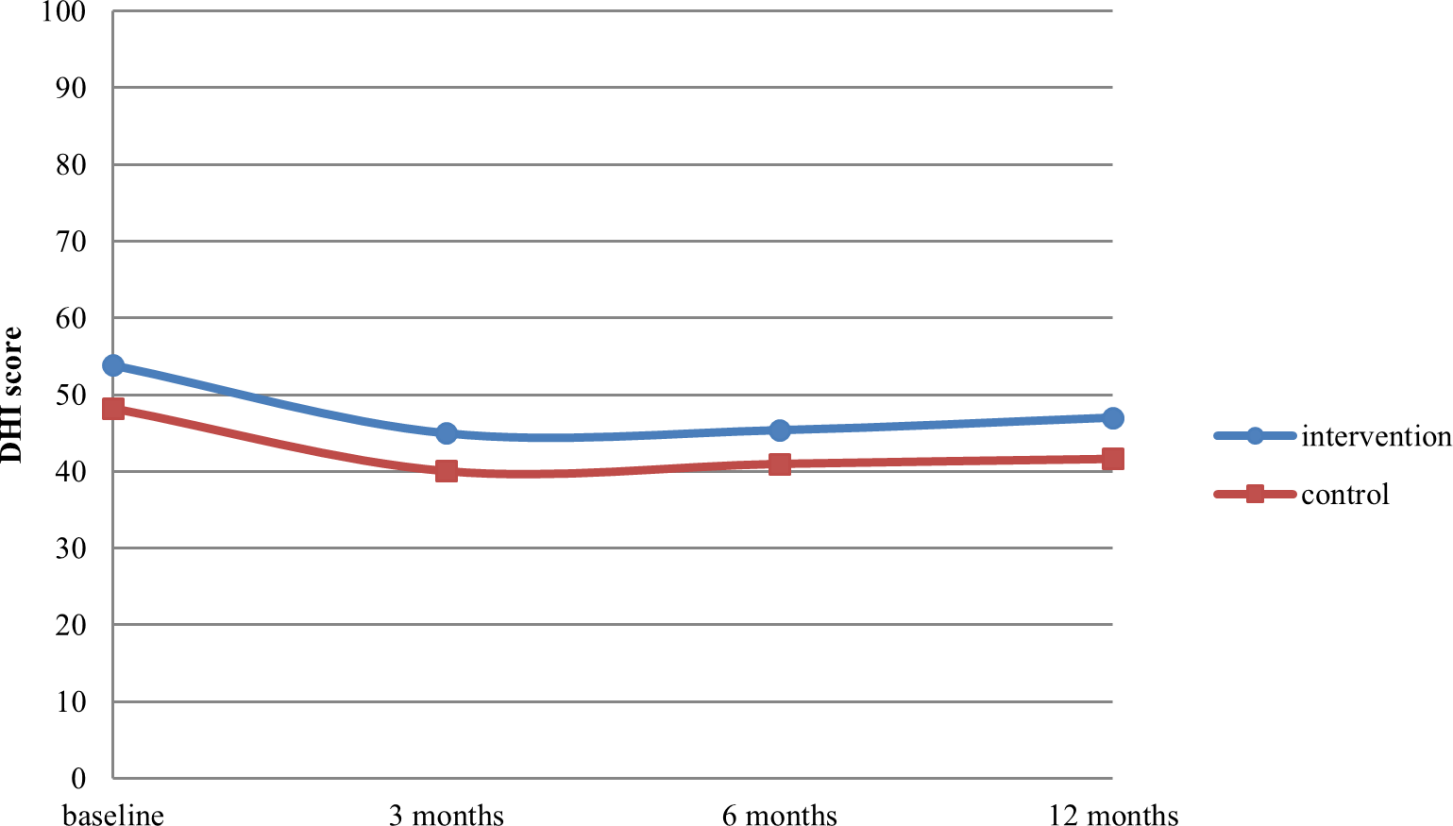
Twelve-month course of dizziness-related impairment as measured with the Dizziness Handicap Inventory (DHI).

We found no significant differences between the intervention group and control group for the primary outcome, DHI score (difference -0.69 [95% CI -5.66;4.28]; p = 0.79). In both groups, the course of the DHI score over time was very similar (Fig 3). An overview of the crude and adjusted overall intervention effects and intervention effects at three, six and 12 months is shown in Tables 2 and 3, respectively. Per protocol analyses did not alter these results (Table 2, per protocol analyses per follow-up measurement not shown).

**Table 2:** Overall intervention effects.

	Intention to treat analysis					Per protocol analysis				
		Crude		Adjusted*			Crude		Adjusted*	
*Continuous outcome measures*	*N*	*Difference (95% CI)*	*P*	*Difference (95% CI)*	*P*	*N*	*Difference (95% CI)*	*P*	*Difference (95% CI)*	*P*
DHI score (0–100)^a^	150	-0.69 (-5.66; 4.28)	0.79	0.65 (-3.97; 5.27)	0.78	126	-2.74 (-8.31; 2.84)	0.34	-1.39 (-6.64; 3.85)	0.60
Dizziness frequency (0–45)^b^	84	-0.26 (-0.84; 0.32)	0.38	-0.32 (-0.91; 0.28)	0.30	71	-0.17 (-0.78; 0.45)	0.60	-0.21 (-0.91; 0.50)	0.57
Quality of life, VAS (0–100)^a^	140	-2.09 (-6.37; 2.18)	0.34	-1.23 (-5.60; 3.25)	0.58	117	-1.43 (-6.21; 3.36)	0.56	-0.60 (-5.62; 4.42)	0.82
Quality of life, utility (-0.29–1.00)^a^	141	0.00 (-0.05; 0.06)	0.94	-0.01 (-0.06; 0.04)	0.60	118	0.00 (-0.05; 0.06)	0.88	-0.02 (-0.07; 0.04)	0.56
Number of FRIDs (0–10)^a^	141	-0.48 (-0.89;-0.06)	**0**.**02**	-0.57 (-1.01;-0.15)	**0**.**01**	119	-0.53 (-1.00;-0.06)	**0**.**03**	-0.67 (-1.18;-0.16)	**0**.**01**
*Dichotomous outcome measures*		*OR (95% CI)*	*P*	*OR (95% CI)*	*P*		*OR (95% CI)*	*P*	*OR (95% CI)*	*P*
Fall frequency^c^	83	1.47 (0.55; 3.89)	0.44	1.07 (0.54; 2.14)	0.84	70	1.78 (0.58; 5.43)	0.31	1.15 (0.54; 2.44)	0.72
Anxiety and depressive disorder^c^	149	1.49 (0.76; 2.92)	0.25	1.49 (0.73; 3.08)	0.28	125	1.73 (0.80; 3.71)	0.16	1.74 (0.74; 4.10)	0.20

N: number of respondents in the analyses; CI: confidence interval; DHI: Dizziness Handicap Inventory; VAS: visual analogue scale; FRID: fall risk increasing drug; OR: odds ratio

^a^ Multilevel linear regression

^b^ Negative binomial regression

^c^ Generalized estimating equation (GEE) logistic regression

* Adjusted for age, sex, living alone, polypharmacy, dizziness onset, dizziness-related impairment, number of comorbidities, anxiety and depressive disorder, cardiovascular disease and functional mobility

**Table 3:** Intervention effects at 3, 6 and 12 months follow-up (intention to treat).

	3 months				6 months				12 months			
	Crude		Adjusted*		Crude		Adjusted*		Crude		Adjusted*	
*Continuous outcome measures*	*Difference (95% CI)*	*P*	*Difference (95% CI)*	*P*	*Difference (95% CI)*	*P*	*Difference (95% CI)*	*P*	*Difference (95% CI)*	*P*	*Difference (95% CI)*	*P*
DHI score (0–100)	-0.04 (-2.70; 4.57)	0.62	1.57 (-3.93; 7.08)	0.57	-1.03 (-6.81; 4.76)	0.73	0.64 (-4.47; 2.83)	0.66	-1.05 (-6.86; 4.77)	0.72	-0.31 (-5.84; 5.22)	0.91
Quality of life, VAS (0–100)	NA		NA		-3.15 (-8.55; 2.25)	0.25	-2.49 (-7.55; 2.57)	0.34	-0.98 (-6.44; 4.48)	0.73	0.15 (-5.00; 5.32)	0.95
Quality of life, utility (-0.29–1.00)	NA		NA		-0.01 (-0.06; 0.05)	0.83	-0.02 (-0.07; 0.03)	0.44	0.01 (-0.05; 0.07)	0.73	-0.00 (-0.06; 0.05)	0.92
*Dichotomous outcome measures*	*OR (95% CI)*	*P*	*OR (95% CI)*	*P*	*OR (95% CI)*	*P*	*OR (95% CI)*	*P*	*OR (95% CI)*	*P*	*OR (95% CI)*	*P*
Anxiety and depressive disorder	1.73 (0.69; 4.32)	0.24	1.78 (0.66; 4.77)	0.25	1.58 (0.66; 3.78)	0.30	1.76 (0.71; 4.38)	0.22	1.25 (0.51; 3.02)	0.63	1.12 (0.42; 3.01)	0.82

CI: confidence interval; DHI: Dizziness Handicap Inventory; VAS: visual analogue scale; OR: odds ratio; NA: not applicable

* Adjusted for age, sex, living alone, polypharmacy, dizziness onset, dizziness-related impairment, number of comorbidities, anxiety and depressive disorder, cardiovascular disease and functional mobility

### Secondary outcomes

No significant intervention effects were found with regard to QoL, dizziness frequency, fall frequency, and anxiety disorder and depression (Tables 2 and 3). The intervention only proved effective in reducing the number of FRIDs (difference -0.48 [95% CI -0.89;-0.06]; p = 0.02). Per protocol analyses showed similar results (Table 2).

## Discussion

### Main findings

The present study aimed to evaluate the effectiveness of a prognosis-oriented approach, i.e. a multifactorial risk factor guided intervention, in older people with dizziness in primary care. We did not find any significant effects of the intervention on the primary outcome, dizziness-related impairment, and secondary outcomes including QoL, dizziness frequency, fall frequency, and anxiety disorder and depression. The intervention significantly reduced the number of FRID prescriptions. Uptake of and adherence to the interventions was significantly lower in patients eligible for two or three interventions compared to patients eligible for one intervention. Refusal and withdrawal were significantly higher for stepped mental health care and exercise therapy, as compared to FRID adjustment.

### Strengths and limitations

This is the first study that investigated the effectiveness of a risk factor guided intervention for dizziness in older patients in primary care, which is a major strength. Other strengths of the study are its randomised-controlled design with a one year follow-up and the inclusion of a vulnerable population (mean age 78.8, significantly impaired, >2 chronic diseases and >7 chronic drugs). Moreover, the pragmatic approach of the study increases the generalizability of our study results to daily practice.

Our study also has some limitations. First, due to time constraints we could only include 168 participants in the study, instead of the 200 participants we planned to include according to our sample size calculation. However, despite the smaller study population there was sufficient power to rule out a clinically relevant difference of 11 DHI points between both groups over time and at the different follow-up measures (95% confidence interval for overall effect on DHI score -5.66 to 4.28) [28]. Second, due to the study design with a multifactorial intervention it is impossible to perform subgroup analyses to assess the effects of the separate components of the multifactorial intervention. Third, uptake of and adherence to the stepped mental health care intervention and the exercise therapy intervention was low. Visiting the mental health nurse practitioner and/or the physiotherapist multiple times in a short period of time might have been too much of a burden for our vulnerable study population.

### Comparison with existing literature

The above-mentioned suboptimal uptake of and adherence to stepped mental health care and exercise therapy is not uncommon with these type of interventions and study population. Previous trials also experienced suboptimal uptake of and adherence to stepped care interventions for depressive symptoms in older adults [38,39], and in frail older patients [40,41]. A systematic review of Sherrington et al. identified 69 trials with exercise interventions to prevent falls in older adults, of which the majority of trials reported moderate to good adherence [42]. However, uptake of the interventions was not reported and in more than half of these 69 trials, participants were younger (average age <75), did not have an increased fall risk, and studied group exercises instead of individual exercise therapy. Finally, the suboptimal uptake of and adherence to the interventions in this study may have been affected by the multifactorial approach, i.e. almost 30% of the participants were eligible for more than one intervention. Potentially, multifactorial treatment may have been too intensive or confusing for the participants. This notion was supported by the fact that uptake of and adherence to the intervention was significantly lower in patients eligible for ≥2 interventions. Uptake of and adherence to FRID medication adjustment, however, was good. This intervention only consisted of one GP visit while the interventions stepped mental health care and exercise therapy required more time and effort from the participants. When it comes to fall prevention interventions in older patients, Nyman et al. showed that multifactorial interventions achieved lower adherence rates than single interventions [43]. Furthermore, Fairhall et al. suggested that single interventions for fall prevention may be equally effective and more easy to implement than multifactorial interventions [44]. Although this study proved to be effective in reducing number of FRIDs, this did not affect dizziness-related impairment. Similarly, other studies with medication interventions in older adults did not find an effect on clinical outcomes such as mortality and falls, even though the interventions also successfully reduced the number of prescriptions [45–47].

### Implications for research and practice

A multifactorial risk factor guided intervention for dizziness in older patients in primary care proved ineffective. Yet, our study adds important insights into multifactorial treatment for dizziness in older people. In this study, several components of the multifactorial intervention proved to be difficult to adhere to for our older participants. Potentially, the separate components of the intervention, and in particular the multifactorial design of the intervention, were too much of a burden for the older study population. Although many researchers suggest multifactorial treatment for older dizzy people [7,19–21,24,48–51], we should reconsider whether this is feasible in daily practice. Instead of multifactorial treatment, future research could focus on a sequential treatment, e.g. measuring effectiveness of various patient-tailored evidence-based therapies in a stepwise approach. Furthermore, it is essential to engage patients in designing future research to increase trial feasibility for the study population.

## Supporting information

S1 Table Consort checklist. (DOCX)

S1 File Translated summary of the guideline ‘Dizziness’ of the Dutch College of General Practitioners (treatment section). (DOCX)

S2 File Study protocol. (PDF)

S3 File Published study. (PDF)
