# Supplementary material for: Effectiveness of a multifactorial intervention for dizziness in older people in primary care: A cluster randomised controlled trial
Source: PLoS One. 2018 Oct 9;13(10):e0204876. doi: 10.1371/journal.pone.0204876 (PMC6178383; doi:10.1371/journal.pone.0204876)
Supplement: S1 File — (DOCX) [file pone.0204876.s002.docx]

**Translated summary of the guideline ‘Dizziness’ of the Dutch College of General Practitioners (treatment section) [1]**

*Information and advice*

- Benign paroxysmal positional vertigo (BPPV). Symptoms of BPPV usually decrease over time within a one month period. The general practitioner (GP) should encourage the patient’s physical activity. If a patient is anxious, the GP can perform the Dix-Hallpike manoeuvre. This will help the patient to relate his symptoms to changes in body position, including the experience that dizziness symptoms will fade away soon after the manoeuvre.
- Vestibular neuronitis. The intensity of dizziness symptoms often makes bed rest necessary. In a number of days the intensity will decrease. However, dizziness symptoms may remain for a longer period of time, although to a lesser extent. There is no treatment option to cure vestibular neuronitis. It may be necessary to prescribe medication to suppress nausea and vomiting.
- Méniѐres disease. Dizziness occurs in attacks, which are very disabling. The dizziness attacks are accompanied with hearing impairment. The frequency of attacks differs between patients. In general, the attacks fade out over the years. Initially, the patient will recover from his hearing impairment after the attack. Yet, over time the hearing impairment will remain. No medication is available to reduce the frequency of attacks, medication to suppress nausea and vomiting may be indicated.
- Orthostatic hypotension. Getting up from a lying or sitting position induces lightheadedness. Some medication potentially induces orthostatic hypotension. Changing this medication sometimes reduces the symptoms of orthostatic hypotension. It is important that the patient slowly gets up form a lying or sitting position and holds on to the bed or chair to avoid falling.

*Non-medicinal treatment*

- BPPV. Brand-Daroff exercises are recommended and the GP might perform the Epley manoeuvre.
- For vestibular neuronitis, the GP should recommend the dizzy patient to keep moving, instead of sitting or lying still.
- For orthostatic hypotension, the GP should give advice on changing the body position and moving around, in order to minimise the symptoms of orthostatic hypotension.
- If orthostatic hypotension a potential side effect of medication use, the GP should evaluate whether this medication can be discontinued.
- In case of dizziness of unknown cause, the GP should focus on functional aspects of dizziness, especially in older patients. Correction of vision (in case of visual impairment), and exercises to improve postural control, fitness, and muscle strength might reduce the feeling of dizziness in older people. Potential consequences of dizziness, like falling and social isolation also require attention.
- Special drugs for dizziness are not recommended, as there is not enough evidence for their effectiveness.

*Evaluation of symptoms*

- BPPV. The patient should visit the GP again after two weeks if symptoms of BPPV do not subside. It is important that the GP searches for other causes of the dizziness symptoms. Yet, if this second check does not yield any leads to other causes, the GP should recommend Brand-Daroff exercises again and perform the Epley manoeuvre.
- Vestibular neuronitis. The patient should visit the GP again after 2-4 days if the symptoms of dizziness do not subside. The GP will perform a neurological exam again to exclude other causes of vertigo.
- Méniѐres disease. The patient should visit the GP in case of a different course of the attack than the patient is used to.
- Patients who discontinued certain medication should have a check-up after 2-4 weeks to evaluate the effect on the dizziness symptoms.
- In case of dizziness of an unknown cause, the patient should visit the GP again after one month if dizziness symptoms persist. Persisting dizziness is often related to anxiety and avoidance. It is important to explicitly ask the patient about anxiety because patients will often not attribute their dizziness to anxiety.

*Referral to specialised care*

- In case of a suspected serious disease, a central neurological cause, or a cardiac cause of dizziness, the patient should be referred to a neurologist or cardiologist.
- In case of an acute peripheral vestibular deficit and hearing complaints the patient should be referred to an ear, nose, and throat (ENT) specialist.
- In case of an unknown cause for dizziness with significant impairment and/or a worried patient, the patient should be referred to a neurologist or ENT specialist.
- In case of suspected Méniѐres disease with diagnostic uncertainty, the GP can refer the patient to an ENT specialist.

*A revised version of the guideline Dizziness is recently published [2]. During inclusion and follow-up of the RODEO study however, the version we translated (version 2002) was applicable.*

**References**

1. Verheij AAA, Van Weert H, Lubbers WJ, Van Sluisveld ILL, Saes GAF, Eizenga WH, et al. The guideline 'Dizziness' of the Dutch College of General Practitioners [in Dutch]. Huisarts Wet. 2002;45:601-9.
2. Bouma M, de Jong J, Dros J, Maarsingh O, Moormann K, Smelt A, et al. The guideline 'Dizziness' of the Dutch College of General Practitioners [in Dutch]. Huisarts Wet. 2017;7:436-9.
