# Supplementary material for: Effectiveness of a multifactorial intervention for dizziness in older people in primary care: A cluster randomised controlled trial
Source: PLoS One. 2018 Oct 9;13(10):e0204876. doi: 10.1371/journal.pone.0204876 (PMC6178383; doi:10.1371/journal.pone.0204876)
Supplement: S2 File — (PDF) [file pone.0204876.s003.pdf]

Improving the prognosis of dizzy elderly: a three-arm validation and intervention study in general practice
(june 2014)

Toetsingonline number	NL49604.029.14
ABR form number	49604
ZonMw project number	839110001
Project number EMGO	WC2014-002
Trial registration number	NTR4346
EudraCT number	n/a

PROTOCOL TITLE 'Improving the prognosis of dizzy elderly: a three-arm validation and intervention study in general practice'

**Protocol ID**	**NL49604.029.14**
**Short title**	**RODEO**<br>**Reduction Of Dizziness in older pEOple**
**Version**	**1**
**Date**	**03-06-2014**
**Coordinating investigator/project leader**	**O.R. Maarsingh (MD, PhD)**<br>**Department of General Practice and Elderly Care Medicine VUmc**<br>**Tel: +31 20 444 52 63**<br>**Fax: +31 20 444 83 61**<br>**E-mail: o.maarsingh@vumc.nl**
**Principal investigator(s)**	**H. Stam (MD)**<br>**Department of General Practice and Elderly Care Medicine VUmc**<br>**Tel: +31 20 444 52 60**<br>**E-mail: stam.h@vumc.nl**
**Sponsor (in Dutch: verrichter/opdrachtgever)**<br><br><br><br><br>***Multicenter research: per site***	**Prof. dr. H.E. van der Horst**<br>**Hoogleraar huisartsgeneeskunde**<br><br>**Head of Department:**<br>**General Practice and Elderly Care Medicine VUm**<br><br>**Sites unknown at this moment**<br>**Follows via amendement after approval of this protocol**
**Independent expert (s)**	**dr. H.W.J. van Marwijk**<br>**Department of General Practice and Elderly Care Medicine VUmc**<br>**Tel: +31 20 444 93 68**<br>**E-mail: HWJ.vanmarwijk@vumc.nl**

PROTOCOL SIGNATURE SHEET

Name	Signature	Date
**Sponsor or legal representative:**<br><br>**Prof. dr. H.E. van der Horst**<br>**Hoogleraar huisartsgeneeskunde**<br><br>**Head of Department:**<br>**General Practice and Elderly Care Medicine VUmc**		
**Principal Investigator:**<br><br>**H. Stam**<br>**PhD Candidate General Medicine and General Practitioner trainee**		

TABLE OF CONTENTS

1. INTRODUCTION AND RATIONALE 9
2. OBJECTIVES 11
3. STUDY DESIGN 12
4. STUDY POPULATION 14
4.1 Population (base) 14
4.2 Inclusion criteria 14
4.3 Exclusion criteria 14
4.4 Sample size calculation 14
5. TREATMENT OF SUBJECTS 17
5.1 Investigational treatment 17
5.2 Use of co-intervention (if applicable) 20
5.3 Escape medication (if applicable) 20
6. INVESTIGATIONAL PRODUCT 21
7. NON-INVESTIGATIONAL PRODUCT 21
8. METHODS 22
8.1 Study parameters/endpoints 22
8.1.1 Main study parameter/endpoint 22
8.1.2 Secondary study parameters/endpoints (if applicable) 22
8.1.3 Other study parameters (if applicable) 22
8.2 Randomisation, blinding and treatment allocation 22
8.3 Study procedures 23
8.4 Withdrawal of individual subjects 25
8.4.1 Specific criteria for withdrawal (if applicable) 26
8.5 Replacement of individual subjects after withdrawal 26
8.6 Follow-up of subjects withdrawn from treatment 26
8.7 Premature termination of the study 26
9. SAFETY REPORTING 27
9.1 Section 10 WMO event 27
9.2 AEs, SAEs and SUSARs 27
9.2.1 Adverse events (AEs) 27
9.2.2 Serious adverse events (SAEs) 27
9.2.3 Suspected unexpected serious adverse reactions (SUSARs) 28
9.3 Annual safety report 28
9.4 Follow-up of adverse events 28
9.5 [Data Safety Monitoring Board (DSMB) / Safety Committee] 28
10. STATISTICAL ANALYSIS 29
10.1 Primary study parameter(s) 29
10.2 Secondary study parameter(s) 29
10.3 Other study parameters 30
10.4 Interim analysis (if applicable) 30
11. ETHICAL CONSIDERATIONS 31

11.1 Regulation statement 31
11.2 Recruitment and consent....31
11.3 Objection by minors or incapacitated subjects (if applicable)....32
11.4 Benefits and risks assessment, group relatedness....32
11.5 Compensation for injury 33
11.6 Incentives (if applicable)....33
12. ADMINISTRATIVE ASPECTS, MONITORING AND PUBLICATION....34
12.1 Handling and storage of data and documents 34
12.2 Monitoring and Quality Assurance....34
12.3 Amendments....34
12.4 Annual progress report....34
12.5 End of study report....34
12.6 Public disclosure and publication policy....35
13. STRUCTURED RISK ANALYSIS 36
14. REFERENCES....37

LIST OF ABBREVIATIONS AND RELEVANT DEFINITIONS

ABR	ABR form, General Assessment and Registration form, is the application form that is required for submission to the accredited Ethics Committee (In Dutch, ABR = Algemene Beoordeling en Registratie)
AE	Adverse Event
AR	Adverse Reaction
CCMO	Central Committee on Research Involving Human Subjects; in Dutch: Centrale Commissie Mensgebonden Onderzoek
CV	Curriculum Vitae
DSMB	Data Safety Monitoring Board
EU	European Union
GCP	Good Clinical Practice
IB	Investigator's Brochure
IC	Informed Consent
METC	Medical research ethics committee (MREC); in Dutch: medisch ethische toetsing commissie (METC)
(S)AE	(Serious) Adverse Event
Sponsor	The sponsor is the party that commissions the organisation or performance of the research, for example a pharmaceutical<br>company, academic hospital, scientific organisation or investigator. A party that provides funding for a study but does not commission it is not regarded as the sponsor, but referred to as a subsidising party.
SUSAR	Suspected Unexpected Serious Adverse Reaction
Wbp	Personal Data Protection Act (in Dutch: Wet Bescherming Persoonsgevens)
WMO	Medical Research Involving Human Subjects Act (in Dutch: Wet Medisch-wetenschappelijk Onderzoek met Mensen

SUMMARY

Rationale: When treating older patients with dizziness in primary care, the current diagnosis oriented approach is insufficient. Often, it is not possible to diagnose an underlying disease and even if a disease is diagnosed, therapeutic options are often limited. In this study a prognosis oriented approach for dizziness in older people in primary care will be investigated.
Objectives: In this study we want to validate a risk score for chronic dizziness with persistent impairment in older dizzy patients in general practice. Furthermore, we want to investigate the effectiveness of a risk factor-guided, i.e. a prognosis-oriented, intervention for dizzy older patients with a high risk of persistent impairment.
Study design: The study protocol consists of two parts. Firstly, we will perform a validation study to externally validate a 7-item risk score for chronic dizziness with persistent impairment. Secondly, we will perform an intervention study, using a cluster controlled clinical trial. In the intervention study we will compare the effectiveness of a targeted intervention for dizzy older patients with a high risk of persistent impairment with usual care during one year of follow-up.
Study population: 375 patients aged 65 years or older who consult their general practitioner (GP) for a new episode of dizziness existing for at least one month.
Intervention: In older patients with a high risk of persistent impairment because of dizziness we will compare a risk factor-guided intervention consisting of one, two, or three of the following interventions: (1) medication adjustment in case of the usage of ≥ 3 Fall Risk Increasing Drugs (FRIDs), (2) stepped care in case of anxiety/depression, and (3) exercise therapy in case of impaired functional mobility. All three interventions are evidence based and usual care for the above-mentioned risk factors.
Main study parameters/endpoints: The primary clinical outcome is dizziness related impairment at follow-up, which will be assessed using the Dizziness Handicap Inventory (DHI). Secondary outcomes are quality of life (EQ-5D-5L), difference in 'Fall Risk Increasing Drug' count, anxiety/depression, dizziness frequency, fall frequency, health care utilization.
Nature and extent of the burden and risks associated with participation, benefit and group relatedness:
A group of 125 patients of the validation group will receive 3 questionnaires by mail at baseline and at 6 and 12 months follow-up. The group of 250 patients participating in the intervention study will receive four questionnaires at baseline, 3, 6 and 12 months follow-up. All 375 patients will be subjected to a baseline assessment of 30 minutes at their home including a structured interview and execution of the Timed 'up-and-go' test. Patients in the intervention group (n=125) will receive – in addition to usual (general practice) care – a targeted intervention, depending on the presence of specific risk-factors. Patients in the

control group (n=125) and validation group (n=125) will receive (general practice) care as usual. No treatment will be denied to any participants nor will it be postponed. There are no major medical health risks for participants in this study. The experimental group may benefit from the intervention by a reduction of impairment due to dizziness and/or a decrease in dizziness frequency.

1. INTRODUCTION AND RATIONALE

Dizziness is one of the ‘geriatric giants’. Thirty percent of people above 65 years of age experience some form of dizziness, increasing to 50% in persons above 85[1]. Nine percent of all persons aged 65 years or older visit their general practitioner (GP) at least once a year because of dizziness. The one-year prevalence of dizziness in general practice is higher in women than in men (65+ years: 9.5% vs. 6.8%) and increases with age (65-74: 6.8%, 75-84: 10.2%; 85+: 10.8%)[2]. Dizziness can lead to persistent limitations in daily functioning and is associated with worsening of depressive symptoms, self-rated health, and a decrease of social activities[3]. Dizziness is one of the most important symptoms negatively affecting well-being in old age[4]. It is also a major risk factor for falling[3,5,6], leading to (fatal and non-fatal) injuries and high healthcare costs[7]. According to Statistics Netherlands (CBS), the annual number of fall related hospital admissions for persons of 65+ years will increase from 43,000 in 2011 to 73,000 in 2030, fall related emergency visits from 83,000 to 140,000, and corresponding healthcare costs from €820 million to €1,400 million[8].

Despite the differences in aetiology of dizziness between younger and older patients[9], guidelines on dizziness advocate the same diagnosis-oriented approach for all patients regardless of their age[10–12]. However, such an approach is insufficient in older patients presenting with dizziness. Often, it is not possible to identify an underlying cause in dizzy older patients. In 40-80% of dizzy older patients, GPs record a symptom diagnosis as the final diagnosis (‘dizziness’ or vertigo’)[2,13]. Moreover, even if a disease is revealed, therapeutic options are limited[14,15]. Therefore, the approach of dizziness in older patients has to change. GPs should not just stick to their diagnostic quest, but also pay attention to the prognosis of a dizzy patient: is this patient at risk of an unfavourable outcome and, if so, how to improve this outcome?[3,15,16]

Several members of our research group initiated the study Dizziness In Elderly Patients (DIEP) in collaboration with the Department of General Practice of the Academic Medical Center Amsterdam in order to fill the gap of knowledge about dizziness in older patients. This study resulted in more knowledge of epidemiology[2,17,18], aetiology[14,19], and diagnosis of dizziness in older patients in general practice[20–23]. Furthermore, a large cohort study investigating the prognosis of dizzy older patients in general practice recently identified seven risk factors for chronic dizziness with persistent impairment[24]. These risk factors are (1) onset of dizziness more than six months ago, (2) dizziness provoked by standing still, (3) trouble with walking and/or falling as associated symptom, (4) polypharmacy, (5) absence of diabetes, (6) anxiety and/or depressive disorder, and (7) impaired functional mobility. Three

of these risk factors are amenable to treatment: polypharmacy, anxiety and/or depressive disorder, and impaired functional mobility.
The results of this cohort study, combined with previous findings of DIEP, provide the basis of the present research proposal.

The first objective of the proposed study is to externally validate our previously developed 7-item risk score for chronic dizziness with persistent impairment[24]. Although the discriminative ability of our developed risk score was good (area under the receiver operating characteristic curve [AUC] of 0.80), it is necessary to validate prediction models in new individuals before they can be implemented in guidelines or applied in clinical practice[25]. If our risk score confirms our previous findings, GPs will have an accurate, easy-to-use clinical tool to identify dizzy older patients with a poor functional prognosis.
Secondly, we want to demonstrate the effectiveness of a risk factor-guided intervention for treating dizziness in older patients in general practice. This intervention includes medication adjustment, a stepped-care program for anxiety/depression, and physical exercise therapy. If proven effective, GPs will be able to improve the functional prognosis of older patients presenting with dizziness, even when a diagnosis is (still) unavailable. Such an intervention will not only improve patients' perceived health but will also lead to a significant reduction of healthcare costs by decreasing fall rate, morbidity (especially fractures) and healthcare utilization. Although several authorities in the field of dizziness – like Mary Tinetti and Philip Sloane – promote a risk factor guided approach of dizziness in older patients[9,15,26], the effectiveness of such an approach has never been investigated.

2. OBJECTIVES

This study aims to investigate if a prognosis-guided clinical strategy is more effective than usual care when treating patients with dizziness in primary care.

Primary Objectives:

1. To validate a previously developed risk score for dizziness-related impairment in older patients in general practice. The risk score consists of seven predictors: 1) onset of dizziness more than six months ago, 2) dizziness provoked by standing still, 3) trouble with walking and/or falling as associated symptom, 4) polypharmacy, 5) absence of diabetes, 6) anxiety and/or depressive disorder, and 7) impaired functional mobility.
2. To investigate the effectiveness of a risk factor-guided intervention for treating dizzy older patients with a high risk of persistent impairment. The intervention includes medication adjustment, a stepped-care program for anxiety/depression, and physical exercise.

Secondary Objectives:

1. To investigate if a targeted intervention in older dizzy patients in general practice will reduce dizziness frequency and fall incidence.
2. To assess if health care utilization of older dizzy patients receiving a targeted intervention will decrease compared to older dizzy patients receiving usual care.
3. To investigate if our targeted intervention is cost-effective.
4. The investigate if older dizzy patients with significant dizziness-related impairment have more often an anxiety- or depressive disorder than non-impaired older dizzy patients in general practice.
5. To investigate the course of anxiety and depression in older dizzy patients in general practice during one year of follow-up.

3. STUDY DESIGN

Both the validation study and the intervention study will be combined in a three-arm design with a follow-up of 1 year. Allocation to the intervention and control condition of the intervention study will be carried out at practice level (cluster randomisation) to avoid contamination.

The figure below represents the flowchart of the study with an overview of the group classification and randomisation. The given percentages are estimates based on secondary analysis of DIEP data[24].

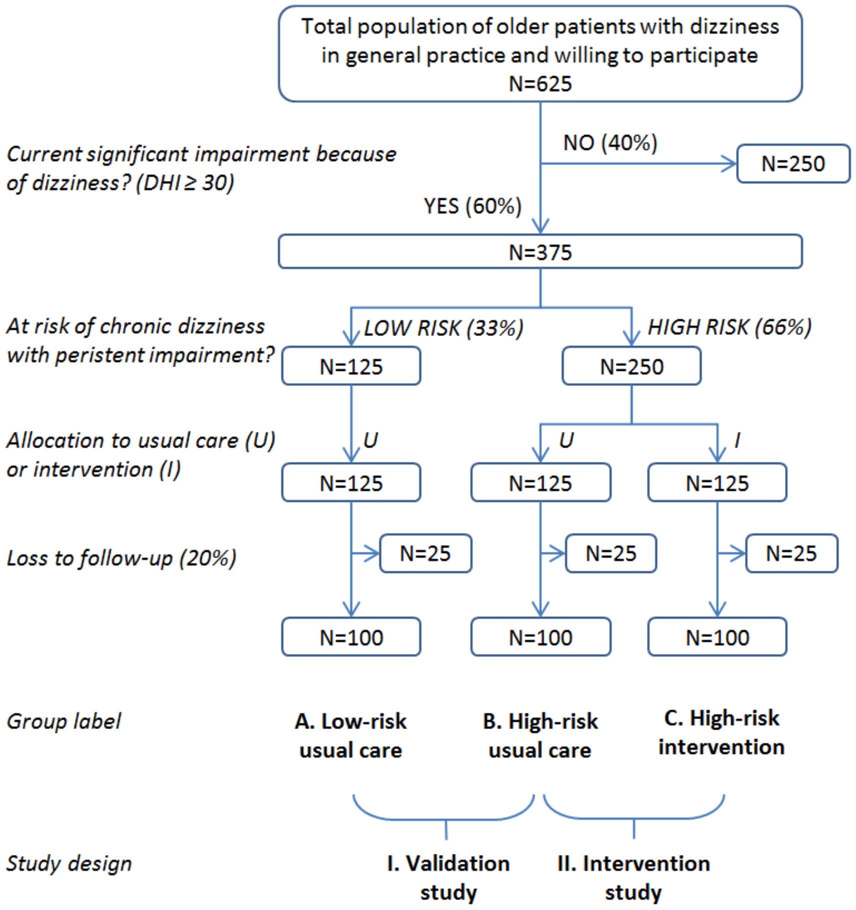

The figure shows that all dizzy older patients will be assigned to group A, B, or C. Assignment to one of the three groups is based on the presence of significant dizziness related impairment at baseline and having a high risk of chronic dizziness with persistent impairment or not. Dizziness related impairment will be assessed with the Dizziness

Handicap Inventory (DHI)[27]. A DHI score of ≥ 30 correlates with current significant impairment because of dizziness. A dizzy older patient will be labelled as high risk for chronic dizziness with persistent impairment in case of a DHI ≥ 30 and the presence of one, two or three of the following risk factors: usage of three or more Fall Risk Increasing Drugs (FRIDs), anxiety and/or depression, and impaired functional mobility.

Summarizing there are three groups: dizzy older patients with significant impairment but at low risk of chronic dizziness with persistent impairment (group A, n=125) and dizzy older people with significant impairment and at high risk of chronic dizziness with persistent impairment (group B and C, n=125 each). Dizzy older people with significant impairment and a high risk of chronic dizziness will be randomised to group B or C (randomisation will take place at practice level). The validation study consists a low risk usual care group (group A) and a high risk usual care group (group B). The intervention study consists of impaired dizzy patients with a ‘high risk’ that will receive usual care (group B) or an intervention (group C).

4. STUDY POPULATION

4.1 Population (base)

This study concerns patients in the primary care setting. Patients will be recruited from the Academic Network of General Practices of the VU University Medical Center (ANH-VUmc) and the General Practitioners Cooperative Twente (Twentse huisartsenonderneming Oost Nederland.

4.2 Inclusion criteria

In order to be eligible to participate in this study, a subject must meet all of the following criteria:

- being 65 years or older
- consulted a general practitioner for a new episode of dizziness, defined as recurrent dizziness for at least one month, including a giddy or rotational sensation, loss of balance, faint feeling, light-headedness, instability, or tendency to fall
- current dizziness-related impairment (DHI ≥ 30)
- ability to speak, read and write Dutch

4.3 Exclusion criteria

A potential subject who meets any of the following criteria will be excluded from participation in this study:

- severe cognitive impairment according to the patient's GP
- serious co morbidity that precludes participation in an exercise program
- current enrolment in interfering study
- if the patient's GP deems participation of the patient in this study undesirable and/or if participation could damage the doctor-patient relationship for any reason. The GP's judgement in relation to possible damage of the doctor-patient relationship will always overrule other in- or exclusion criteria.

4.4 Sample size calculation

Intervention study

The sample size calculation for our intervention study is based on the difference in 1-year DHI score change between the high-risk intervention group and the high-risk usual care group. We consider a DHI score difference of 12 or more to be a clinically relevant difference[28]. The mean DHI score of patients above 65 consulting their GP because of dizziness is 36.3 with a standard deviation of 19.9[14]. In order to detect a clinically relevant

difference with alpha=0.01 and beta=0.90 we used the following formula to calculate the sample size[29]:

$$n = 2(2.58+1.28)2*S2/D^2$$

With n = number of patients, S = standard deviation, D = clinically relevant difference
A group size of n=82 is sufficient $(2(2.58+1.64)^2 \times 19.92^2/12^2)$[29]. However, we need to apply an extra correction since the proposed study is a cluster randomised trial. The correction that needs to be applied by performing an cluster randomised trial can be calculated with the following formula:

$$n = 1+(k-1) * \rho$$

With n = number of patients, k = cluster size, ρ = intra-class correlation (ICC)
Based on secondary analysis of DIEP data we expect a cluster size of 5 patients per practice[14]. An ICC of 0.01 is often used in sample size calculation in primary care research[30]. However, we used an ICC of 0.05 to get a more conservative sample size. The correction factor for performing a cluster randomised trial is 1.2 (1+(5-1) * 0.05). This means that a groups size of n = 99 (1.2*82) is sufficient. Taking into account a loss to follow-up of 20% we need 124 participants in each arm. Thus, a total of 250 patients will be included in the intervention study.

Validation study
The aim of the validation study is to validate a previously developed risk-score[24]. This risk-score consists of 7 predictors and was constructed to predict the risk of chronic dizziness with persistent impairment (this means a DHI score ≥ 30 at 6 months follow-up).
A commonly used rule of thumb to judge sample sizes for developing diagnostic and prognostic models is the recommendation of at least 10 events per variable. This rule is based on estimates of the stability of coefficient estimates for individual variables in the model. According to this rule, at 6 months follow-up at least 7*10 = 70 patients need to suffer from dizziness with a DHI-score ≥ 30 to enable validation of the risk-score. Based on secondary analysis of DIEP data we expect that 60% of the patients with a DHI ≥ 30 at baseline will have a DHI score of ≥ 30 at 6 months follow-up[14]. Additionally to the validation of the previous developed risk score, we anticipate to model some 10-15 candidate variables to construct a new risk-score. With sample sizes of 125 patients in group A and 125 patients in group B we will be able validate the previous developed risk

score and we will be able to construct a new risk score with 15 predictors in impaired dizzy patients (0.6 * 250 = 150, 150/10 = 15).

5. TREATMENT OF SUBJECTS

5.1 Investigational treatment

All patients of the intervention group (group C, n=125) will receive one, two or three interventions. The interventions are based on three treatable risk factors for chronic dizziness with persistent impairment. The three risk factors are: usage of ≥ 3 Fall Risk Increasing Drugs (FRIDs), anxiety/depression, and impaired functional mobility.
Medication adjustment will be offered in case of the usage of ≥ 3 FRIDs, stepped-care will be offered in case of anxiety/depression and exercise therapy will be given in case of impaired functional mobility. All three interventions are evidence based and usual care for the above-mentioned risk factors.
Based on secondary analysis of DIEP data[14], we estimate that 62% of patients in the intervention group will be eligible to receive medication adjustment, 48% to receive stepped-care, and 37% to receive exercise therapy. Additionally, we estimate that 62% of patients in the intervention group will be eligible to receive only one intervention, 28% to receive two interventions, and 9% to receive all three interventions.

1. Medication adjustment

Dizzy patients using ≥ 3 FRIDs will be subjected to a FRID-analysis[31]. The list of FRIDs include psychotropic drugs (sedatives, antidepressants, neuroleptics), cardiovascular drugs (antihypertensives, nitrates, anti-arrhythmics, nicotinic acid, β-adrenoceptor blocker eye drops) and other drugs (analgesics, antivertigo preparations, hypoglycaemics, urinary antispasmodics). Interventional studies demonstrated a reduction in falls and postural instability following FRID-withdrawal[31,32].
All study patients will be asked about medication use during baseline assessment. After baseline assessment current prescribed medication will be requested from the patients pharmacist. The medication list will be sent by fax to the researcher. The patient will receive a phone call by the researcher for extra clarification if there is a discrepancy between the medication list supplied by the patient and the medication list supplied by the pharmacist.
Once a month, a trained pharmacist and an independent GP will have a meeting to review the FRID use of all patients of the intervention ‘medication adjustment’ that have been included in the preceding month. In every individual patient of group C, all potential FRIDs will be considered for withdrawal. If a FRID is considered redundant, the pharmacist and GP will advise to stop it. If a FRID is considered necessary, the pharmacist and GP will advise to change to another drug that sorts the same effect, or to reduce the dosage if considered safely possible. The results of the FRID-analysis, including considerations for

discontinuation/withdrawal of FRIDs, will be handed to the patients GP and practice nurse by fax. The practice nurse will invite the patient for a consultation with his/her GP within two weeks. The GP will discuss the outcomes of the FRID-analysis with the patient and will stop or withdrawal the FRIDs if the patient agrees. The GP will fill out a small questionnaire to give feedback about adjustment of medication to researcher.
If discontinuation or withdrawal of FRID appears to be impossible, the GP and the patient will receive a letter of the researcher.

2. Stepped-care for anxiety/depression

Dizzy older patients with the risk factor ‘anxiety and/or depression’ (defined as the presence of Generalised Anxiety Disorder (GAD), Panic Disorder (PD), or Major Depressive Disorder (MDD)), will follow a stepped-care program, according to the Trimbos Multidisciplinary Guideline Anxiety Disorders[33] and the Trimbos Multidisciplinary Guideline Depression[34]. The presence of GAD/PD/MDD will be assessed by the and GAD-7, Patient Health Questionnaire, panic module (PHQ-PD), and the PHQ-9 (see next paragraph). Stepped-care has been proven to be effective in older people with anxiety and/or depression[35,36].
Stepped-care involves four subsequent treatment steps for anxiety and depression in primary care. A practice nurse mental health (POH-GGZ) of the patient’s own general practice guides the patient through the various steps of the program. All participating POH’s-GGZ will receive a training of two days about stepped-care, guided self-help and problem solving therapy before start of the intervention. The GP is easily accessible for the POH-GGZ to discuss about a patient if needed. The steps of the program involve: (1) watchful waiting, 6 weeks; (2) bibliotherapy, 6 weeks; (3) Problem Solving Therapy, 6 weeks; and (4) indicated treatment. The patient will proceed to the next step after every 6 weeks in case of persisting complaints. The POH-GGZ will seek for an alternative treatment step if a patient is not motivated for an offered intervention. The intervention, as part of our study, is finished when the patient does not suffer from anxiety/depression anymore, or when the patient still suffers from anxiety/depression and proceeds to step 4.
Step 1. Watchful waiting. The first 6 weeks consists of a period of watchful waiting, because it is known that depressive/anxiety symptoms often disappear spontaneously over time. In this 6 week period, the patient will be invited by the POH-GGZ for a first consultation to get familiar with the POH-GGZ. During this introductory consultation the POH-GGZ will shortly inform the patient about depression/anxiety and the stepped-care program. After 6 weeks the POH-GGZ will invite the patient again. The patient will be asked to fill out the GAD-7, PHQ-PD and PHQ-9. In case of persisting anxiety and/or depression the patient will go to step 2.

Step 2. Bibliotherapy. During this step the patient will start with a guided self-help course (based on Lewinsohn's 'Coping with Depression' course[37]). This self-help course is based on cognitive behavioural techniques and takes 6 weeks. The patient will receive a course book and every week, the patient will read about new insights and skills to cope with anxiety and/or depression. The book consists of information and exercises, which the patient can work through at his/her own pace. The POH-GGZ will support the patient by contacting the patient by telephone every two weeks, and by providing extra guidance if necessary. Since many patients of our population will be visual impaired, the POH-GGZ will also receive a digital version of the course to be able to print the text in a large size if necessary. After 6 weeks the POH-GGZ will invite the patient. The POH-GGZ will discuss the current depressive/anxiety symptoms and the patient will be asked to fill out the GAD-7, PHQ-PD and PHQ-9 again. In case of persisting anxiety and/or depression the patient will go to step 3.

Step 3. Problem Solving Treatment (PST). PST is a brief cognitive behavioural intervention that focuses on practical skill building, education and managing depressive symptoms. The goal is to achieve a reduction in mental health problems by stimulating an active attitude towards everyday problems[38]. PST will be offered by the POH-GGZ and takes a maximum of 7 sessions at the general practice.

At the end of the PST-course the POH-GGZ will discuss the current depressive/anxiety symptoms and will ask the patient to fill out the GAD-7, PHQ-PD and PHQ-9 again. If a depression and/or anxiety still exists the patient is offered to go to step 4.

Step 4. Indicated treatment. The POH-GGZ will discuss the outcomes of the previous treatment steps with the GP if the patient is still suffering from anxiety and/or depression after the preceding steps. The GP will then have a consultation with the patient to assess what would be an appropriate next therapy for the patient (e.g. starting with antidepressants). The GP will then initiate the treatment.

3. Exercise therapy for risk factor impaired functional mobility

Dizzy elderly with the risk factor 'impaired functional mobility' (defined as a TUG score of 20 seconds or more[39]), will receive standardized exercise therapy by a physiotherapist. Earlier research has shown that exercise therapy aiming to improve strength and balance in older people has a positive effect on mobility and reduces rate of falls and risk of falling[40–43]. The exercise therapy implies training in a group session of one hour twice a week for eight weeks and will focus on strength (including resistance) and balance.

The program starts with 15 minutes warming up exercises in standing and walking with different combinations of head, trunk, arm and leg movements. This will be followed by a circuit training with 8 stations. The patients will perform a two minute exercise at every

station and will perform two laps of the circuit. The stations include the following exercises: standing on foam with eyes closed, standing on foam while flexing the knees and turning the head from side to side, a walking exercise with slalom between cones, knee flexor/extensor training with an ankle cuff, hip abductor/adductor training with an ankle cuff, standing up on toes, and standing on heels. All exercise of the circuit will be individually tailored by performing the exercises with or without extra support to remain standing, varying with the weight of the ankle cuff, standing or walking on heels and toes and standing or jumping on the trampoline. The exercise therapy will end with a cooling down of 10 minutes. The program will be accompanied by music of the group's preference[44,45].

No adverse reactions other than musculoskeletal discomfort related to exercise therapy has been described, in particularly no fall related injuries occurring during exercise programs have been described[42,46].

Usual care

All included patients of group A and B (n=125) will receive usual care by their GPs. Participating GPs will receive a written instruction, asking them to diagnose causes of dizziness according to the Dutch guideline on dizziness[11] and to treat identified disorders according to the guidelines of the Dutch College of GPs[47].

5.2 Use of co-intervention (if applicable)

Not applicable.

5.3 Escape medication (if applicable)

Not applicable.

6. INVESTIGATIONAL PRODUCT

Not applicable.

7. NON-INVESTIGATIONAL PRODUCT

Not applicable.

8. METHODS

8.1 Study parameters/endpoints

8.1.1 Main study parameter/endpoint

The primary clinical outcome is dizziness related impairment, which will be assessed using the DHI[27]. For the validation study we will dichotomize all DHI scores. In the intervention study we will use the difference in the 1-year DHI score change between patients in the intervention group and patients in the control group.

8.1.2 Secondary study parameters/endpoints (if applicable)

- Quality of life, as measured with the EQ-5D-5L.
- Dizziness frequency, defined as the number of episodes of dizziness per day, week, month and year. Dizziness frequency will be assessed using a calendar filled in by the patient during a 12 month period.
- Difference in FRID count, defined as the difference in FRID count between baseline and after 12 months follow-up.
- Presence of anxiety/depression, as measured with GAD-7, PHQ-PD, and PHQ-9.
- Fall frequency, defined as the number of falls per year. Fall incidence will be assessed using a calendar filled in by the patient during a 12 month period.
- Health care utilization. We will use the extracted data from the electronic medical records of all patients to assess the number of registered medical consultations, prescriptions, referrals, hospital admissions, and nursing home admissions.

8.1.3 Other study parameters (if applicable)

Not applicable.

8.2 Randomisation, blinding and treatment allocation

Randomisation will take place at practice level. Randomisation will be executed by a researcher who is not involved in the selection of the practices (concealment of allocation). Practices will be randomly assigned to intervention practice or control practice. Stratification will take place for practice type (solo or group practice) and area (practice in disadvantaged areas or not) and block randomization will be used to create similar distributions in the different study arms. Randomisation of practices will be executed before inclusion of patients.

8.3 Study procedures

Questionnaires

If a patient agrees to participate in the study he/she will receive a set of questionnaires. Patients in group A (n = 125) will receive three questionnaires at baseline and at 6 and 12 months follow-up. Patients in the intervention study (group B and C, n = 250) will receive four questionnaires at baseline and at 3, 6 and 12 months follow-up The questionnaires will be discussed later in this section.

Baseline assessment

All patients will be subjected to a baseline assessment of 30 minutes during a home visit or at the surgery of the patient's GP, depending on the preference of the patient and the mobility of the patient. Baseline assessment will be carried out by a physician or a trained research assistant.

The baseline assessment includes a structured interview and the TUG.

Structured interview. This includes record of socio-demographic characteristics, intoxications, current medication use, history of falls, dizziness characteristics, and the use of a hearing, seeing or walking aid.

Timed 'up and go' Test (TUG)[39]. The TUG will be executed to assess impaired functional mobility. The TUG consists of an observation of the patient rising from a standard arm chair (approximate seat height of 46 cm), walking to a line on the floor 3 meters away, turning, returning, and sitting down again. The subject wears his regular footwear and uses his customary walking aid (none, cane or walker). No physical assistance is given. The patient starts with his/her back against the chair with his/her arms resting on the chair's arms, and his/her walking aid at hand. On the word 'go' the patient gets up and walks at a comfortable and safe pace. The patient will walk through the test once before being timed in order to become familiar with the test. A test score of ≥ 20 seconds reflects impaired functional mobility[39].

Structured interviews

In order to estimate the effect of attention in the intervention group, a random sample of 30 patients in the high risk usual care group (group B) will receive an additional home visit from a medical student, during which structured interviews will be conducted. This structured interview will take about 1 hour and will be about the actual impact of dizziness on the patient's everyday life.

The estimated effect of attention will be used in the final analysis of the results of the intervention study.

Outcome measures

Questionnaires

- Dizziness handicap inventory (DHI)[27].
  Time to fill out: 10 minutes.
  The DHI is a widespread used self-report questionnaire, designed to quantify the impact of dizziness on everyday life. The DHI has 25 items with 3 answer categories ('yes', 'sometimes', or 'no'), investigating self-perceived physical, emotional, and functional disability associated with dizziness. DHI scores range from 0-100. A higher DHI score indicates a higher level of dizziness-related impairment. A DHI score of ≥ 30 correlates with current significant impairment because of dizziness.
- Dizziness handicap inventory, short version (DHI-S)[48].
  This is a short screening version of the DHI, in which 10 items of the original DHI are extracted. The 10 items in the DHI-S are scored are also scored in 3 answer categories ('yes', 'sometimes', or 'no'). A DHI score of ≥ 12 correlates with current significant impairment because of dizziness.
- Generalised Anxiety Disorder-7 (GAD-7)[49,50].
  Time to fill out: 5 minutes.
  The GAD-7 is a 7-item questionnaire to identify GAD. Scores on the GAD-7 range from 0 to 21; scores of 5, 10, and 15 represent mild, moderate, and severe anxiety symptoms, respectively.
- Patient Health Questionnaire, panic module (PHQ-PD)[51].
  Time to fill out: 5 minutes.
  The PHQ-PD will enable us to determine the presence of PD. The diagnostic algorithm for PD consists of 15 questions with 2 answer categories (yes or no) and is considered fulfilled if the answer to questions 3a-d is ''yes'' and if the answer to 4 or more of questions 4a-k is ''yes''[51].
- 9-item Patient Health Questionnaire depression module (PHQ-9)[52,53].
  Time to fill out: 5 minutes.
  The PHQ-9 is designed to detect depression. The PHQ-9 has 9 questions with a score ranging from 0 to 3 for each question (maximum score of 27). A threshold score of 10 or higher is considered to indicate mild MDD, 15 or higher indicates moderate MDD, and 20 or higher severe MDD.
- Euro Quality of Life–5-dimension, 5-level (EQ-5D-5L)[54].

Time to fill out: several minutes.

The EQ-5D-5L is a questionnaire that defines health in five dimensions (mobility, self-care, usual activities, pain/discomfort, anxiety/depression) and is often used to translate the effect of an intervention into Quality Adjusted Life Years (QALY's)[54].

Calendar

- Dizziness and fall calendar for patients of group B and C (n = 250). At baseline assessment the patient will be handed out a calendar to keep up frequency dizziness and falls for 12 months. The patient will be instructed in the use of keeping up dizziness and fall frequency with the calendar. The patient will be asked to fill out the calendar every week which will take no more than 1 minute weekly.

The following table gives an overview of all measurements and instruments.

Overview of all measurements and instruments

	Validation study			Intervention study			
	baseline	6 mo.	12 mo.	baseline	3 mo.	6 mo.	12 mo.
Screening dizziness handicap (DHI-S)	x			x			
Baseline assessment	x			x			
Dizziness Handicap (DHI)	x	x	x	x	x	x	x
QoL (EQ-5D-5L)	x	x	x	x		x	x
Depression (PHQ-9)	x	x	x	x	x	x	x
Anxiety (GAD-7)	x	x	x	x	x	x	x
Panic disorder (PHQ-PD)	x	x	x	x	x	x	x
FRID count	x		x	x			x
Dizziness and fall frequency (calendar))				------------------ weekly --------------------			

8.4 Withdrawal of individual subjects

Subjects can leave the study at any time for any reason if they wish to do so without any consequences. The investigator can decide to withdraw a subject from the study for urgent medical reasons.

8.4.1 Specific criteria for withdrawal (if applicable)

Patients will be withdrawn from the study if they are diagnosed with severe cognitive impairment. Furthermore, patients participating in the intervention-arm ‘exercise therapy’ will be withdrawn from the intervention if the patients develops serious comorbid conditions that precludes participation in an exercise program.

8.5 Replacement of individual subjects after withdrawal

Patients will be asked to participate in the study until the sample-size for the study has been reached. Patients will not be replaced from intervention group to control group or vice versa.

8.6 Follow-up of subjects withdrawn from treatment

Patients who have withdrawn from treatment will be asked to keep up filling out the questionnaires since we aim to perform an intention-to-treat analysis. Furthermore, reason for drop out will be stored in the database to be able to perform a loss-to follow-up analysis if necessary.

8.7 Premature termination of the study

Not applicable.

9. SAFETY REPORTING

9.1 Section 10 WMO event

In accordance to section 10, subsection 1, of the WMO, the investigator will inform the subjects and the reviewing accredited METC if anything occurs, on the basis of which it appears that the disadvantages of participation may be significantly greater than was foreseen in the research proposal. The study will be suspended pending further review by the accredited METC, except insofar as suspension would jeopardise the subjects' health. The investigator will take care that all subjects are kept informed.

9.2 AEs, SAEs and SUSARs

9.2.1 Adverse events (AEs)

Adverse events are defined as any undesirable experience occurring to a subject during the study, whether or not considered related to the experimental intervention. All adverse events reported spontaneously by the subject or observed by the investigator or his staff will be recorded.

9.2.2 Serious adverse events (SAEs)

A serious adverse event is any untoward medical occurrence or effect that at any treatment:

- results in death;
- is life threatening (at the time of the event);
- requires hospitalisation or prolongation of existing inpatients' hospitalisation;
- results in persistent or significant disability or incapacity;
- Any other important medical event that may not result in death, be life threatening, or require hospitalization, may be considered a serious adverse experience when, based upon appropriate medical judgement, the event may jeopardize the subject or may require an intervention to prevent one of the outcomes listed above.

Procedures for handling the serious adverse events.

The sponsor will report the SAEs through the web portal *ToetsingOnline* to the accredited METC that approved the protocol, within 15 days after the sponsor has first knowledge of the serious adverse events.

SAEs that result in death or are life threatening will be reported expedited. The expedited reporting will occur not later than 7 days after the responsible investigator

has first knowledge of the adverse event. This is for a preliminary report with another 8 days for completion of the report.

9.2.3 Suspected unexpected serious adverse reactions (SUSARs)

Not applicable.

9.3 Annual safety report

Not applicable.

9.4 Follow-up of adverse events

All AEs will be followed until they have abated, or until a stable situation has been reached. Depending on the event, follow up may require additional tests or medical procedures as indicated, and/or referral to the general physician or a medical specialist.

SAEs need to be reported till end of study within the Netherlands, as defined in the protocol.

9.5 [Data Safety Monitoring Board (DSMB) / Safety Committee]

Not applicable.

10. STATISTICAL ANALYSIS

General characteristics of dizzy and control participants will be described quantitatively. The data derived from questionnaires and the fall calendar will also be presented in a quantitative way. Missing data will be handled with multiple imputation analysis[55].

10.1 Primary study parameter(s)

I. Validation study

The external validity of our 7-item risk score will be quantified by assessing the reliability, discrimination and calibration of the model[25]. The reliability of the model will be quantified with the Hosmer-Lemeshow goodness-of-fit statistic. We will assess the calibration of the model by plotting the predicted probabilities against the observed frequencies of persistent dizziness-related impairment. We will assess the discriminative ability of the model, i.e. its ability to distinguish dizzy patients with persistent dizziness-related impairment from dizzy patients without persistent dizziness-related impairment, by calculating the receiver operating characteristic curve (AUC). An AUC of 0.5 indicates no discrimination above chance, whereas an AUC of 1.0 indicates perfect discrimination. We will use R statistical software for bootstrapping to adjust for over-optimism in model performance.

II. Intervention study

We will perform both intention-to-treat and per-protocol analysis. We will use univariate and multivariate analyses to compare outcomes of usual care and intervention group. To investigate the effectiveness of the risk factor-guided intervention, we will use a repeated-measures mixed-effect (RMME) model to assess differences in the 1-year DHI score change, adjusted for potential confounders.

10.2 Secondary study parameter(s)

Presence of anxiety/depression, quality of life, dizziness frequency, fall incidence and health care utilization will be taken along in the univariate and multivariate analysis as stated above.

The economic evaluation will be analysed according to the intention-to-treat principle. Costs typically have a highly skewed distribution. Therefore, bootstrapping with 5000 replications will be used to calculate 95% confidence intervals around the mean difference in total costs between the treatment groups. The effect measure that will be used for the cost-effectiveness is EQ-5D-5L. Incremental cost-effectiveness ratios (ICERs) will be calculated by dividing the difference in mean total costs between the

treatment groups by the difference in mean effects between the treatment groups. Bootstrapping will be used to estimate the uncertainty surrounding the ICERs which will be graphically presented on cost-effectiveness planes. Cost-effectiveness acceptability curves and net monetary benefits will also be calculated. Cost-effectiveness acceptability curves show the probability that the intervention is cost-effective in comparison with usual care for a range of different ceiling ratios thereby showing decision uncertainty.

10.3 Other study parameters

Not applicable.

10.4 Interim analysis (if applicable)

No interim analysis will be carried out since this study has a follow-up of only one year.

11. ETHICAL CONSIDERATIONS

11.1 Regulation statement

This study will be conducted according to the principles of the Declaration of Helsinki (Version October 2013, adopted at the 64th WMA General Assembly, Fortaleza, Brazil) and in accordance with the Medical Research Involving Human Subjects Act (WMO).

11.2 Recruitment and consent

Practices

This study will be embedded in the Academic Network of General Practices of the VU Medical Center (ANH-VUmc, www.anh-vumc.nl) and the General Practitioners Cooperative Twente (Twentse huisartsenonderneming Oost Nederland, www.thoon.org).
After randomisation, all GP's in the participating practices will be informed about the outcome (intervention practice or control practice) of the randomisation. At this point the GP's will also receive extra information about the study and instructions for the GPs, specified to the situation of the practice (intervention or control practice). The following care professionals will be invited for trainings: pharmacists who will participate in group meetings for FRID-analysis; physiotherapists for the physiotherapy intervention; and POH-GGZs and psychologists who will participate in the stepped-care program.

Recruitment of patients

Patients will be recruited via two routes: through identification of patients by the GPs and via searches in the electronic medical databases (EMD) of the GPs. We will use the strategy of identification of patients by the GP since this has been proven to be successful in the DIEP study. In addition to this we will ask the GP's to do a search in their EMDs every three months to make sure we will not miss eligible patients.
Identification of patients by the GPs will take place during consultation hours. If a GP sees an eligible patient for this study, the GP will inform the patient about the presence of the study and will ask for the patients' permission to be approached by the researcher. If the patient has given his/her permission, the GP will register the obtained permission in the EMD and inform the researcher (by fax or e-mail) about the patient's name and address. The researcher will then send the patient an invitation letter.
Secondly, the GP will be asked to execute a search in his/her EMD every three months. The GP will exclude all identified dizzy patients that fulfil the exclusion criteria. The researcher will then check which patients have not yet been invited to participate in the

study. Patients identified through searches in the EMD will receive an invitation letter on behalf of their GP.

Additionally to the invitation letter, all patients will receive a brochure with study information, a contact form and the DHI-S. Patients will be asked to fill out the DHI-S if they want to participate in the study. All patients with a DHI-S of ≥ 12 will receive a phone call of the researcher to set an appointment for baseline assessment at the patients home. Patients with a DHI-S score of < 12 are not eligible to participate in the study.

Consent

Informed consent will be obtained in two steps; by a contact form and an informed consent form. Firstly, the patient receives contact form together with the invitation letter. If the patient decides to be willing to participate in the study then he/she will sign the contact form. By signing the contact form, the patient gives permission to be approached by the researcher.

About two weeks later, before the start of the baseline assessment at the patients home, the informed consent procedure including signing of the informed consent form will take place. The patient has had sufficient time to think about participating in the study at this point and there is room for the patient to ask questions to the researcher or research assistant. By signing the informed consent form, the patient agrees to participate in the study.

11.3 Objection by minors or incapacitated subjects (if applicable)

Not applicable.

11.4 Benefits and risks assessment, group relatedness

This study aims to provide GPs with an easy-to-use clinical tool to identify dizzy older patients with a poor functional prognosis. The identification of dizzy elderly with a poor functional prognosis will enable the GP to provide better care to these patients. Furthermore the investigation of the effectiveness of a risk factor guided intervention for dizzy older patients might result in less impairment due to dizziness and/or a decrease in dizziness frequency in older patients.

To assess the clinical tool, the effectiveness of the intervention and the cost-effectiveness of the intervention it is necessary to collect data via baseline assessment and questionnaires.

There are no major medical health risks for participants in this study. The experimental group might benefit from their extra therapy by possibly resulting in less impairment due to dizziness and/or a decrease in dizziness frequency.

11.5 Compensation for injury

The sponsor/investigator has a liability insurance which is in accordance with article 7, subsection 9 of the WMO.

The sponsor (also) has an insurance which is in accordance with the legal requirements in the Netherlands (Article 7 WMO and the Measure regarding Compulsory Insurance for Clinical Research in Humans of 23th June 2003). This insurance provides cover for damage to research subjects through injury or death caused by the study.

1. € 450.000,-- (i.e. four hundred and fifty thousand Euro) for death or injury for each subject who participates in the Research;
2. € 3.500.000,-- (i.e. three million five hundred thousand Euro) for death or injury for all subjects who participate in the Research;
3. € 5.000.000,-- (i.e. five million Euro) for the total damage incurred by the organisation for all damage disclosed by scientific research for the Sponsor as 'verrichter' in the meaning of said Act in each year of insurance coverage.

The insurance applies to the damage that becomes apparent during the study or within 4 years after the end of the study.

11.6 Incentives

Not applicable.

12. ADMINISTRATIVE ASPECTS, MONITORING AND PUBLICATION

12.1 Handling and storage of data and documents

Information from questionnaires will be inserted in a structural database. During baseline assessment all data will immediately be registered in Blaise, a software programme for standardized data registration that helps to avoid the occurrence of missing data. Data from Blaise can be extracted and collected in the structural database later. We will use subject identification numbers and the key to the code of the subject identification number is safeguarded by the principal investigator. Only the principal investigator and the research assistants will have access to the source data. Data will be kept for as long as the study takes, and at least 5 years.

12.2 Monitoring and Quality Assurance

An independent monitor from the Clinical Research Bureau (CRB) of VUmc will have access to the data and source documents of the trial. Monitoring will be performed in compliance with Good Clinical Practice (GCP) and other rules and regulations in order to achieve high quality research and secure patient safety. Detailed monitoring procedures will be described by the CRB in a study-specific monitoring plan.

12.3 Amendments

Amendments are changes made to the research after a favourable opinion by the accredited METC has been given. All amendments will be notified to the METC that gave a favourable opinion.

12.4 Annual progress report

The sponsor/investigator will submit a summary of the progress of the trial to the accredited METC once a year. Information will be provided on the date of inclusion of the first subject, numbers of subjects included and numbers of subjects that have completed the trial, serious adverse events/ serious adverse reactions, other problems, and amendments.

12.5 End of study report

The investigator will notify the accredited METC of the end of the study within a period of 8 weeks. The end of the study is defined as the last patient’s last visit.

In case the study is ended prematurely, the investigator will notify the accredited METC within 15 days, including the reasons for the premature termination.

Within one year after the end of the study, the investigator/sponsor will submit a final study report with the results of the study, including any publications/abstracts of the study, to the accredited METC.

12.6 Public disclosure and publication policy

The results of research will be submitted for publication to peer-reviewed scientific journals. There are no restrictions placed upon publication by the sponsor of this study (*ZonMw, programma Kwaliteit van Zorg: Versnellen, verbreden, vernieuwen*).

13. STRUCTURED RISK ANALYSIS

Not applicable.

14. REFERENCES

1. Jönsson R, Sixt E, Landahl S, Rosenhall U. Prevalence of dizziness and vertigo in an urban elderly population. *J Vestib Res*. 2004;14(1):47–52.

2. Maarsingh OR, Dros J, Schellevis FG, van Weert HC, Bindels PJ, Horst HE Van Der. Dizziness reported by elderly patients in family practice: prevalence, incidence, and clinical characteristics. *BMC Fam Pract*. 2010;11(2).

3. Tinetti ME, Williams CS, Gill TM. Health, functional, and psychological outcomes among older persons with chronic dizziness. *J Am Geriatr Soc*. 2000;48(4):417–21.

4. Grimby A, Rosenhall U. Health-related quality of life and dizziness in old age. *Gerontology*. 1995;41(5):286–98.

5. Pluijm SMF, Smit JH, Tromp EAM, et al. A risk profile for identifying community-dwelling elderly with a high risk of recurrent falling: results of a 3-year prospective study. *Osteoporos Int*. 2006;17(3):417–25.

6. Luukinen H, Koski K, Kivela SL, Laippala P. Social status, life changes, housing conditions, health, functional abilities and life-style as risk factors for recurrent falls among the home-dwelling elderly. *Public Health*. 1996;110(2):115–8.

7. Hartholt KA, Polinder S, Van der Cammen TJM, et al. Costs of falls in an ageing population: a nationwide study from the Netherlands (2007-2009). *Injury*. 2012;43(7):1199–203.

8. Valongevallen 65-plussers. Available at: http://www.veiligheid.nl/cijfers/valongevallen-65-plussers.

9. Sloane PD, Coeytaux RR, Beck RS, Dallara J. Dizziness: state of the science. *Ann Intern Med*. 2001;134(9 Pt 2):823–32.

10. Fife TD, Tusa RJ, Furman JM, et al. Assessment: Vestibular testing techniques in adults and children: Report of the Therapeutics and Technology Assessment Subcommittee of the American Academy of Neurology. *Neurology*. 2000;55(10):1431–1441.

11. Verheij A, Van Weert H, Lubbers W, et al. The guideline “Dizziness” of the Dutch College of General Practitioners. *Huisarts Wet*. 2002;45:601–609.

12. Huh Y-E, Kim J-S. Bedside evaluation of dizzy patients. *J Clin Neurol*. 2013;9(4):203–13.

13. Kruschinski C, Kersting M, Breull A, Kochen MM, Koschack J, Hummers-Pradier E. Diagnosehäufigkeiten und Verordnungen bei Schwindel im Patientenkollektiv einer hausärztlichen Routinedatenbank. *Z Evid Fortbild Qual Gesundhwes*. 2008;102(5):313–319.

14. Maarsingh OR, Dros J, Schellevis FG, et al. Causes of Persistent Dizziness in Elderly Patients in Primary Care. *Ann Fam Med*. 2010;8(3):196–205.

15. Tinetti ME, Williams CS, Gill TM. Dizziness among older adults: a possible geriatric syndrome. *Ann Intern Med*. 2000;132(5):337–44.

16. Bailey KE, Sloane PD, Mitchell M, Preisser J. Which primary care patients with dizziness will develop persistent impairment? *Arch Fam Med*. 1993;2(8):847–52.

17. Dros J, Maarsingh OR, Beem L, et al. Impact of dizziness on everyday life in older primary care patients: a cross-sectional study. *Health Qual Life Outcomes*. 2011;9(1):44.

18. Maarsingh OR, Schellevis FG, van der Horst HE. Looks vestibular: irrational prescribing of antivertiginous drugs for older dizzy patients in general practice. *Br J Gen Pract*. 2012;62(603):518–20.

19. Dros J, Maarsingh OR, van der Windt D a WM, et al. Profiling dizziness in older primary care patients: an empirical study. *PLoS One*. 2011;6(1).

20. Dros J, Maarsingh OR, van der Horst HE, Bindels PJ, Ter Riet G, van Weert HC. Tests used to evaluate dizziness in primary care. *CMAJ*. 2010;182(13):E621–31.

21. Maarsingh OR, Dros J, van der Windt DA, et al. Diagnostic indicators of anxiety and depression in older dizzy patients in primary care. *J Geriatr Psychiatry Neurol*. 2011;24(2):98–107.

22. Dros J, Maarsingh OR, van der Windt DA. Diagnostic test results in older patients with and without dizziness: a primary care based case-control study. *Submitted*. 2013.

23. Maarsingh OR, Dros J, van Weert HC, Schellevis FG, Bindels PJ, van der Horst HE. Development of a diagnostic protocol for dizziness in elderly patients in general practice: a Delphi procedure. *BMC Fam Pract*. 2009;7(10):12.

24. Dros J, Maarsingh OR, Beem L, et al. Functional prognosis of dizziness in older adults in primary care: a prospective cohort study. *J Am Geriatr Soc*. 2012;60(12):2263–9.

25. Moons KGM, Kengne AP, Grobbee DE, et al. Risk prediction models: II. External validation, model updating, and impact assessment. *Heart*. 2012;98(9):691–8.

26. Kao AC, Nanda A, Williams CS, Tinetti ME. Validation of dizziness as a possible geriatric syndrome. *J Am Geriatr Soc*. 2001;49(1):72–5.

27. Jacobson GP, Newman CW. The development of the Dizziness Handicap Inventory. *Arch Otolaryngol Head Neck Surg*. 1990;116(4):424–7.

28. Tamber A-L, Wilhelmsen KT, Strand LI. Measurement properties of the Dizziness Handicap Inventory by cross-sectional and longitudinal designs. *Health Qual Life Outcomes*. 2009;7:101.

29. Florey CD. Sample size for beginners. *BMJ*. 1993;306(6886):1181–4.

30. Adams G, Gulliford MC, Ukoumunne OC, Eldridge S, Chinn S, Campbell MJ. Patterns of intra-cluster correlation from primary care research to inform study design and analysis. *J Clin Epidemiol*. 2004;57(8):785–94.

31. Bennett DA, Gnjidic D, Gillett M, et al. Prevalence and impact of fall-risk-increasing drugs, polypharmacy, and drug-drug interactions in robust versus frail hospitalised falls patients: a prospective cohort study. *Drugs and Aging*. 2014;31(3):225–32.

32. Van der Velde N, Stricker BHC, Pols H a P, van der Cammen TJM. Risk of falls after withdrawal of fall-risk-increasing drugs: a prospective cohort study. *Br J Clin Pharmacol*. 2007;63(2):232–7.

33. Van der Velde N, van den Meiracker AH, Pols H a P, Stricker BHC, van der Cammen TJM. Withdrawal of fall-risk-increasing drugs in older persons: effect on tilt-table test outcomes. *J Am Geriatr Soc*. 2007;55(5):734–9.

34. Balkom A van, Vliet I van, Emmelkamp P, et al. Multidisciplinary Guideline Anxiety Disorders (third revision). 2013. Available at: http://www.ggzrichtlijnen.nl/.

35. Spijker J, Bockting C, Meeuwissen J, et al. Multidisciplinary Guideline Depression (third revision). 2013. Available at: http://www.ggzrichtlijnen.nl/.

36. Van ’t Veer-Tazelaar P, van Marwijk H, van Oppen P, et al. Stepped-care prevention of anxiety and depression in late life: a randomized controlled trial. *Arch Gen Psychiatry*. 2009;66(3):297–304.

37. Van ’t Veer-Tazelaar P, van Marwijk H, van Oppen P, et al. Prevention of late-life anxiety and depression has sustained effects over 24 months: a pragmatic randomized trial. *Am J Geriatr Psychiatry*. 2011;19(3):230–9.

38. Cuijpers P, Muñoz RF, Clarke GN, Lewinsohn PM. Psychoeducational treatment and prevention of depression: the “Coping with Depression” course thirty years later. *Clin Psychol Rev*. 2009;29(5):449–58. doi:10.1016/j.cpr.2009.04.005.

39. Mynors-Wallis L, Davies I, Gray A, Barbour F, Gath D. A randomised controlled trial and cost analysis of problem-solving treatment for emotional disorders given by community nurses in primary care. *Br J Psychiatry*. 1997;170(Feb):113–9.

40. Podsiadlo D, Richardson S. The timed “Up & Go”: a test of basic functional mobility for frail elderly persons. *J Am Geriatr Soc*. 1991;39(2):142–8.

41. Gillespie LD, Robertson MC, Gillespie WJ, et al. Interventions for preventing falls in older people living in the community. *Cochrane database Syst Rev*. 2012;9:CD007146.

42. Howe TE, Rochester L, Neil F, Skelton DA, Ballinger C. Exercise for improving balance in older people. *Cochrane database Syst Rev*. 2011;(11):CD004963.

43. El-Khoury F, Cassou B, Charles M-A, Dargent-Molina P. The effect of fall prevention exercise programmes on fall induced injuries in community dwelling older adults: systematic review and meta-analysis of randomised controlled trials. *BMJ*. 2013;347(October):f6234.

44. De Vries NM, van Ravensberg CD, Hobbelen JSM, Olde Rikkert MGM, Staal JB, Nijhuis-van der Sanden MWG. Effects of physical exercise therapy on mobility, physical functioning, physical activity and quality of life in community-dwelling older

adults with impaired mobility, physical disability and/or multi-morbidity: a meta-analysis. *Ageing Res Rev*. 2012;11(2):136–49.

45. Gardner MM, Buchner DM, Robertson MC, Campbell a J. Practical implementation of an exercise-based falls prevention programme. *Age Ageing*. 2001;30(1):77–83.

46. Kammerlind AS, Håkansson JK, Skogsberg MC. Effects of balance training in elderly people with nonperipheral vertigo and unsteadiness. *Clin Rehabil*. 2001;15(5):463–70.

47. Gardner MM, Robertson MC, Campbell a J. Exercise in preventing falls and fall related injuries in older people: a review of randomised controlled trials. *Br J Sports Med*. 2000;34(1):7–17.

48. Guidelines of the Dutch College of General Practitioners. Available at: https://www.nhg.org/nhg-standaarden.

49. Jacobson GP, Calder JH. A screening version of the Dizziness Handicap Inventory (DHI-S). *Am J Otol*. 1998;19(6):804–8.

50. Arroll B, Goodyear-Smith F, Crengle S, et al. Validation of PHQ-2 and PHQ-9 to screen for major depression in the primary care population. *Ann Fam Med*. 2010;8(4):348–53.

51. Phelan E, Williams B, Meeker K, et al. A study of the diagnostic accuracy of the PHQ-9 in primary care elderly. *BMC Fam Pract*. 2010;11:63.

52. Spitzer RL, Kroenke K, Williams JBW, Löwe B. A brief measure for assessing generalized anxiety disorder: the GAD-7. *Arch Intern Med*. 2006;166(10):1092–7.

53. Kroenke K, Spitzer RL, Williams JBW, Löwe B. The Patient Health Questionnaire Somatic, Anxiety, and Depressive Symptom Scales: a systematic review. *Gen Hosp Psychiatry*. 2010;32(4):345–59.

54. EuroQol Group. Available at: http://www.euroqol.org/.

55. Donders ART, van der Heijden GJ, Stijnen T, Moons KG. Review: A gentel introduction to imputation of missing values. *J Clin Epidemiol*. 2006;59(10):1087–1091.
