# Supplementary material for: Effectiveness of a multifactorial intervention for dizziness in older people in primary care: A cluster randomised controlled trial
Source: PLoS One. 2018 Oct 9;13(10):e0204876. doi: 10.1371/journal.pone.0204876 (PMC6178383; doi:10.1371/journal.pone.0204876)
Supplement: S3 File — (PDF) [file pone.0204876.s004.pdf]

Stam *et al. Trials* (2015) 16:313
DOI 10.1186/s13063-015-0848-1

TRIALS

STUDY PROTOCOL Open Access

Impairment reduction in older dizzy people in primary care: study protocol for a cluster randomised controlled trial

Hanneke Stam*, Johannes C. van der Wouden, Henriëtte E. van der Horst and Otto R. Maarsingh

Abstract

Background: The management of dizziness in older patients is primarily diagnosis-oriented. However, in 40 % of older patients with dizziness, GPs are not able to identify an underlying cause, and a number of common underlying causes of dizziness cannot (or hardly) be treated. In this study we will investigate the effectiveness of a prognosis-oriented approach in the management of dizziness in older patients. This prognosis-oriented approach comprises identification of patients at risk for chronic dizziness with persistent impairment by identifying risk factors for an unfavourable course of dizziness. Patients at risk for chronic dizziness with persistent impairment will be offered treatment addressing the identified modifiable risk factors.

Methods/Design: This study will be performed in primary care. An intervention study and a validation study will be conducted in a three-arm cluster randomised design. In the intervention study we will investigate a risk factor guided multi-component intervention. The risk factor guided intervention includes: (1) medication adjustment in case of three or more prescribed fall-risk-increasing drugs, (2) stepped care in case of anxiety disorder and/or depression, and (3) exercise therapy in case of impaired functional mobility. The primary outcome measure is dizziness-related impairment, which will be assessed with the Dizziness Handicap Inventory. Secondary outcome measures are quality of life, anxiety disorder and depression, use of fall-risk-increasing drugs, dizziness frequency, fall frequency, and healthcare utilization.

Discussion: This study is, to date, the first study that will investigate the effectiveness of a prognosis-oriented approach for reducing dizziness-related impairment in older people in primary care. Offering treatment that addresses identified modifiable risk factors to patients at high risk for chronic dizziness is unique. The pragmatic design of this study will enable evaluation of the outcomes in real-life routine practice conditions. An effective intervention will not only reduce dizziness-related impairment, but may also decrease healthcare utilization and healthcare costs. The previously developed risk score that will be validated alongside the intervention study will enable GPs to identify patients at high risk for chronic dizziness with persistent impairment.

Trial registration: Netherlands Trial Register (identifier: NTR4346), registration date 15 December 2013.

Keywords: Dizziness, Aged, Prognosis, Therapy, Quality of life, Impairment

* Correspondence: stam.h@vumc.nl
Department of General Practice and Elderly Care Medicine and EMGO Institute for Health and Care Research, VU University Medical Center, Van der Boechorststraat 7, room D550, 1081 BT Amsterdam, The Netherlands

BioMed Central

© 2015 Stam et al. This is an Open Access article distributed under the terms of the Creative Commons Attribution License (http://creativecommons.org/licenses/by/4.0), which permits unrestricted use, distribution, and reproduction in any medium, provided the original work is properly credited. The Creative Commons Public Domain Dedication waiver (http://creativecommons.org/publicdomain/zero/1.0/) applies to the data made available in this article, unless otherwise stated.

Background

Dizziness occurs frequently in older people. The prevalence of dizziness in older people ranges from 8 % in the primary care population to 30 % in the community, and increases with age [1–7]. For doctors, dizziness is a challenging entity to deal with: patients use the term dizziness for a variety of sensations, and the complaint dizziness may accompany harmless but also very serious conditions. Dizziness is a fuzzy concept that can refer to several sensations, including a giddy or rotational sensation, a loss of balance, a faint feeling, light-headedness, instability or unsteadiness, a tendency to fall, or a feeling of everything turning black [8]. Dizziness in older people can have serious consequences. Dros et al. reported that more than 60 % of older dizzy people in primary care experience moderate or severe impact on daily living due to dizziness [9]. Furthermore, dizziness is associated with worsening of depressive symptoms, self-rated health, and social activities [10]. Older people who experience dizziness also have an increased fall risk [10], leading to injury and high healthcare costs [11]. Hartholt et al. showed that 3 % of all Dutch people aged ≥65 years yearly visit the emergency department due to falling, resulting in a mean cost of €9370 per fall, increasing to €14,600 per fall in patients aged ≥80 years [11].

Most guidelines on dizziness tend to advocate a diagnosis-oriented approach regardless of the age of the patient [12, 13]. The diagnosis-oriented approach starts with a search for the cause of dizziness, and treatment follows once an illness is diagnosed. For several reasons, this diagnosis-oriented approach may be insufficient in older patients presenting with dizziness. Firstly, in 40 % of older patients with dizziness GPs are not able to identify an underlying cause [1]. Secondly, a number of common underlying causes of dizziness cannot be treated (such as polyneuropathy) or hardly treated (such as orthostatic hypotension). Finally, clinicians may identify causes of dizziness for which treatment is available but not desirable (such as the Epley manoeuvre for benign paroxysmal position vertigo in patients with severe cervical arthrosis). Croft et al. recently stated that many illnesses cannot usefully be labelled from a disease-diagnosis perspective [14]. They argued that in such cases, a prognostic model can provide an alternative framework for clinical practice that extends beyond disease and diagnosis, and incorporates a wide range of information to predict future patient outcomes and to guide decisions to improve them [14].

Tinetti et al. suggested that dizziness in the aged may constitute a multifactorial geriatric syndrome [4, 15]. Geriatric syndromes, such as delirium, falls, incontinence, and frailty, are highly prevalent, multifactorial, and associated with substantial morbidity and poor outcomes [16]. Research on contributory factors for dizziness in older people has strengthened the idea of dizziness in the aged as being a geriatric syndrome [17–21]. Several researchers presume that a multifactorial intervention, targeting contributory factors of the geriatric dizziness syndrome, might reduce dizziness and dizziness-related impairment [4, 8, 15, 17, 18, 21]. To date, the effectiveness of such a multifactorial approach has not been investigated. In this study, aged patients with dizziness will be offered a multi-component intervention in a so-called prognosis-oriented approach. This prognosis-oriented approach focuses on the identification of modifiable risk factors for an unfavourable course of dizziness, after which treatment will be offered that addresses the modifiable risk factors [22].

The aim of this study is to evaluate the effectiveness of a prognosis-oriented approach in older people with dizziness in primary care. We will perform an intervention study to investigate the effectiveness of a risk factor-guided intervention for treating dizziness in older patients. This risk factor-guided intervention consists of the following interventions: (1) medication adjustment in case of ≥3 prescribed fall-risk-increasing drugs (FRIDs), (2) stepped care in case of anxiety and/or depression, and (3) exercise therapy in case of impaired functional mobility. Alongside this intervention study we will validate a previously developed seven-item risk score for chronic dizziness with persistent impairment in older people [19].

Methods/Design

The study has a three-arm design to perform both an intervention study and a validation study with a 1-year prospective follow-up period (Fig. 1). The intervention study is a cluster randomized controlled trial, designed to investigate the effectiveness of a risk factor-guided multi-component intervention for treating dizziness in older patients. The aim of the validation study is to validate a previously developed risk score for chronic dizziness with persistent impairment in older people in primary care [19]. Patients will be assigned to one of three study groups: the intervention group (Fig. 1, group A), the observational cohort (Fig. 1, group B), and the control group (Fig. 1, group C). In the intervention study, the intervention group and the control group will be compared. The validation study comprises the observational cohort and the control group.

The study was approved by the Medical Ethics Review Committee of VU University Medical Center (approval number: NL49604.029.14), and will be conducted according to the principles of the Declaration of Helsinki (version 2013) and the Dutch Medical Research Involving Human Subjects Act (WMO). For this paper, we followed the Consolidated Standards of Reporting Trials

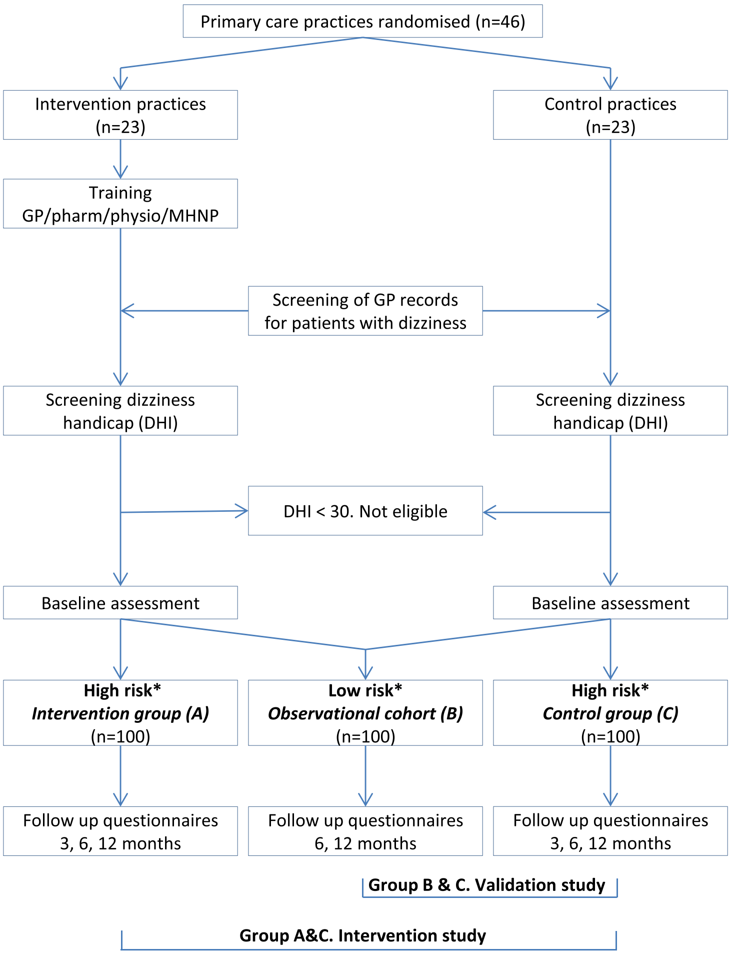

Fig. 1 Flow chart of the study. * High risk or low risk of chronic dizziness with persistent impairment. *DHI* Dizziness Handicap Inventory, *MHNP* mental health nurse practitioner, *GP* general practitioner, *pharm* pharmacologist, *physio* physiotherapist

(CONSORT) statement with extension to cluster randomised trials [23].

Sample and setting

Patients will be recruited from 46 general practices in an urban area in the Netherlands. Patients of 65 years and older are eligible to participate in the study if they have consulted their general practitioner (GP) for dizziness in the preceding 3 months, and if they are significantly impaired by their dizziness. We define dizziness as recurrent dizziness for at least 1 month, including a giddy or rotational sensation, loss of balance, faint feeling, lightheadedness, instability, and/or tendency to fall [8]. The Dizziness Handicap Inventory (DHI) will be used to assess impairment due to dizziness. A DHI score of ≥30 corresponds with significant impairment due to dizziness [24]. Exclusion criteria are severe cognitive impairment, terminal illness, severe psychiatric problems, and the inability to speak, read and write Dutch.

Randomisation

In the intervention study, cluster randomisation at practice level will be conducted to avoid contamination. General practices will be randomised by a researcher who is blinded to their identity (concealment of allocation) before the inclusion of patients begins. Practices will be stratified by list size into practices with up to 400, 400 to 800, and over 800 patients of 65 years and older. For each stratum, block randomisation with varying block size will be used to create similar distributions in the study arms. The investigator is also blinded to the size of each block.

Sample size calculation

We intend to include a total of 300 subjects. The sample size calculation for our intervention study is based on the difference in change in 1-year DHI score between the intervention group and the control group. We consider a DHI score difference of 11 or more to be clinically

relevant [25]. With an α of 0.05, a β of 0.20, an intraclass correlation coefficient of 0.05, and the assumption of a loss to follow-up of 20 %, a sample of 100 patients in both the intervention and control group is needed.

To calculate the sample size of the validation study, a commonly used rule of thumb is followed that at least 10 events per variable are available for developing diagnostic and prognostic models. The previously developed risk score that will be validated consists of seven predictors and estimates the functional prognosis of dizziness at 6 months. With the assumption that 60 % of the patients with a DHI score ≥30 at baseline will have a DHI score of ≥30 after 6 months of follow-up [26], a sample of 100 patients in both the observational cohort and the control group will enable validation of the previously developed risk score.

Recruitment

Patients will be recruited by the GPs via two routes. Firstly, GPs will invite patients to participate in the study during consultation hours. Secondly, every 3 months older patients with dizziness will be identified by searching the electronic medical records (EMRs) in all participating general practices. The search in the EMRs will be executed by searching for International Classification of Primary Care (ICPC) codes N17 (dizziness) and H82 (vertiginous syndromes), and free text words dizz*, vertig*, and the Dutch equivalent for 'dizz'.

All patients will receive an invitation letter from their GP including study information and the DHI to assess impairment due to dizziness. Patients interested in participating will be asked to send back a completed DHI to the researcher. The researcher will contact all patients with a DHI score of ≥30 to arrange an appointment for baseline assessment at the patient's home. Before the start of baseline assessment, written informed consent will be obtained from each participant.

Assignment to study groups

Assignment to one of the three study groups (Fig. 1) is based on the risk of chronic dizziness with persistent impairment, which will be assessed during baseline assessment at the patient's home. An older patient with dizziness will be labelled as having a high risk for chronic dizziness with persistent impairment if they have a DHI score of ≥30 at baseline and the presence of one, two, or three of the following risk factors: (1) three or more prescribed FRIDs, (2) anxiety disorder and/or depression, and (3) impaired functional mobility.

Patients with a high risk of chronic dizziness will participate in the intervention group or control group, depending on whether their general practice is an intervention practice or control practice respectively. Older patients with dizziness with significant impairment, but at low risk of chronic dizziness with persistent impairment, will be allocated to the observational cohort.

In sum, the intervention study consists of a control group with patients with dizziness-related impairment and a high risk of chronic dizziness that will receive usual care (Fig. 1, group C), and an intervention group that receives usual care plus one, two, or three interventions (Fig. 1, group A). In sum, the intervention study consists of impaired dizzy patients with a 'high risk' that will receive usual care in the control group (Fig. 1, group C) and usual care plus 1, 2, or 3 interventions in the intervention group (Fig. 1, group C).

Interventions

All patients of the intervention group (n = 100) will receive usual care plus one, two, or three risk factor-guided interventions. The offered interventions are: (1) medication adjustment in case of three or more prescribed FRIDs; (2) stepped care in case of anxiety disorder and/or depression; and (3) exercise therapy in case of impaired functional mobility. Patients eligible for more than one intervention will start the applicable interventions at the same time.

Medication adjustment in case of three or more fall-risk-increasing drugs

This intervention will be similar to the 'FRID withdrawal' study by Van der Velde et al. [27, 28]. The list of FRIDs includes psychotropic drugs (sedatives, antidepressants, and neuroleptics), cardiovascular drugs (antihypertensives, nitrates, anti-arrhythmics, nicotinic acid, and β-adrenoceptor blocker eye drops), and other drugs (analgesics, anti-vertiginous drugs, hypoglycaemics, and urinary antispasmodics) [27]. Once a month, a pharmacist and an independent GP will have a meeting to review the FRID use of all patients eligible for the intervention 'medication adjustment' that have been included in the preceding month. In every individual patient, all potential FRIDs will be considered for withdrawal. The use of FRIDs will be stopped if no health risks are involved, or reduced in dose when stopping is not an option. The FRID medication advice will be handed to the patient's GP, who will be asked to invite the patient on his or her consultation hour to discuss the FRID medication advice. FRID medication adjustment will only take place if both the GP and the patient agree.

Stepped care for anxiety disorder and/or depression

Older patients with dizziness in the intervention group with the risk factor 'anxiety disorder and/or depression' (defined as the presence of generalised anxiety disorder (GAD), panic disorder (PD) or major depressive disorder (MDD)), will be offered a stepped care program, based

on the Trimbos Multidisciplinary Guideline Anxiety Disorders [29] and the Trimbos Multidisciplinary Guideline Depression [30]. The presence of GAD, PD, and/or MDD will be assessed by the Generalised Anxiety Disorder-7 (GAD-7) [31], Patient Health Questionnaire Panic Module (PHQ-PD) [32], and Patient Health Questionnaire-9 (PHQ-9) [33, 34], respectively.

The offered stepped-care program involves four subsequent evidence-based treatment steps for anxiety disorder and depression in primary care, lasting 6 weeks each. A mental health nurse-practitioner (MHNP) working in the patient's own general practice will guide the patient through the steps of the program. All participating MHNPs will receive a training before the start of the intervention. If needed, the GP will be available for the MHNP to discuss a patient. The patient flow through the stepped care program depends on the patient's symptom level, as measured with the GAD-7, PHQ-PD, and PHQ-9 at the end of every step. Patients who still have elevated anxiety or depression symptom scores (a GAD-7 score ≥10, a PHQ-9 score ≥10, or a positive PHQ-PD score) after concluding a step will be invited to participate in the next step. The steps are listed below:

Step 1 (watchful waiting): The first 6 weeks consists of watchful waiting. At the start of step one the MHNP will invite the patient for an introductory consultation. The patient will get acquainted with the MHNP and the MHNP will inform the patient about the stepped-care program. No therapy will be offered during step one.

Step 2 (guided self-help treatment): During this step the patient will start with a guided self-help course of 6 weeks. The course is based on Lewinsohn's 'Coping with Depression' course [35] and modified for patients of 65 years and over who suffer from anxiety disorder and/or depression. The course includes of six modules that last a week each. The patient will read about new insights and skills and is challenged to perform exercises. The consecutive modules focus on acknowledgment of being anxious or depressed, relaxation, pleasant activity scheduling, changing of cognitions, and assertiveness. The MHNP will call the patient every 2 weeks.

Step 3 (problem-solving treatment; PST): PST is a brief cognitive behavioural intervention that focuses on practical skill building, education, and managing anxiety and/or depression symptoms. The goal is to reduce mental health problems by stimulating an active attitude towards everyday problems [36]. PST will be offered by the MHNP and takes a maximum of six sessions of 30 min at the GP surgery. The stages of problem-solving are explained during the sessions and then applied to problems that are encountered in daily life.

Step 4 (referral to the GP): The GP will have a consultation with the patient to assess what would be an appropriate next therapy for the patient (for example, starting with antidepressants or referral to a psychologist). The GP will then initiate the treatment.

Exercise therapy in case of impaired functional mobility

Older patients with dizziness-related impairment in the intervention group with the risk factor 'impaired functional mobility' (defined as a Timed Up-and-Go score of 20 s or more [37]), will receive standardized exercise therapy by a physiotherapist of the patient's choice. Physical exercise therapy has a positive effect on mobility and physical functioning in mobility limited and/or physically disabled older patients [38]. We choose for an individual intervention because the effect of individual interventions seems to be somewhat larger than the effect of group interventions [38]. The aim of exercise therapy is to improve resistance and balance because this might improve strength and balance, which are often implicated as a cause of mobility impairment [39]. The intervention implies training of one hour twice a week for 8 weeks. Every training session starts with exercises on sitting balance, static standing balance, and trunk stability. The program then continues with seated leg press, cable lat pulldown, cycling, and concludes with walking on the treadmill. The physiotherapists will receive study information and a treatment protocol that is designed together with a senior physiotherapist specialised in exercise programs. The treatment protocol prescribes what exercises should be carried out every week and includes pictures of the specific exercises. The treatment protocol is designed to give individualised care: the intensity of the exercise therapy will be adjusted according to the patients' physical condition that will be tested during the intake.

Usual care

Patients in the control group and the observational cohort will have unrestricted access to usual care: no treatment will be denied to any participants nor will it be postponed. The GPs of control practices will not be informed about the intervention and will not receive any training. Instead, they will be asked to provide care as recommended in the guideline 'Dizziness' of the Dutch College of General Practitioners [13].

Primary outcome measure

All primary and secondary outcome measures are summed up in Table 1. The intervention group and control group will be followed up on at 3, 6, and 12 months;

Table 1 Overview of measurements and instruments

	Validation study			Intervention study			
	baseline	6 months	12 months	baseline	3 months	6 months	12 months
Baseline assessment	x			x			
Dizziness Handicap (DHI)	x	x	x	x	x	x	x
QoL (EQ-5D-5L)	x	x	x	x		x	x
Depression (PHQ-9)	x	x	x	x	x	x	x
Anxiety (GAD-7)	x	x	x	x	x	x	x
Health care utilisation (EMR)			x				x
Panic disorder (PHQ-PD)	x	x	x	x	x	x	x
FRID count	x		x	x			x
Dizziness and fall frequency (calendar)				——————— weekly ———————			

DHI dizziness handicap inventory, *EMR* electronic medical records, *FRID* Fall-risk-increasing drug, *GAD-7* Generalised Anxiety Disorder-7, *PHQ-9* Patient Health Questionnaire-9, *QoL* Quality of Life, *PHQ-PD* Patient Health Questionnaire Panic Module

the observational cohort will be followed up on at 6 and 12 months.

The primary clinical outcome is dizziness-related impairment, which will be assessed using the DHI [24]. The DHI is a widely used self-report questionnaire, designed to quantify the impact of dizziness on everyday life, with scores ranging from 0 to 100. A DHI score of ≥30 correlates with current significant impairment because of dizziness [24]. In the intervention study, the primary outcome is the difference in change in 1-year DHI score between patients in the intervention group and patients in the control group. For the validation study, we will dichotomise all DHI scores (<30 or ≥30).

Secondary outcome measures

Secondary outcome measures that will be measured in both the intervention study and the validation study are quality of life, presence of anxiety disorder and/or depression, and healthcare utilisation. Furthermore, in the intervention study dizziness frequency, fall frequency, and difference in FRID count will also be measured.

Quality of life, will be measured with the Euro Quality of Life-5-dimension, 5-level (EQ-5D-5L) [40]. The EQ-5D-5L is a questionnaire that assesses health in five dimensions (mobility, self-care, usual activities, pain and/or discomfort, and anxiety and/or depression).

Presence of anxiety disorder and/or depression will be measured with GAD-7, PHQ-PD, and PHQ-9 [31–34].

Healthcare utilization: after informed consent, we will extract data from the general practitioners' EMRs of all participating patients to assess the number of consultations, prescriptions, referrals, hospital admissions, and nursing home admissions.

Dizziness frequency and fall frequency, defined as the number of episodes of dizziness and falls per day, week, month, and year, will be assessed using a calendar filled in by the patient during a 12-month period. The calendar is similar to a fall calendar used in an earlier study [41], and is designed to monitor dizziness frequency additionally to fall frequency.

Difference in FRID count, defined as the difference in number of prescribed FRIDs between baseline and at 12 months follow-up, will also be measured.

Statistical analysis

Intervention study

We will use descriptive statistics to describe the study population. Dropout and loss to follow-up will be described. The primary analysis will be an intention-to-treat analysis. We will also perform a per-protocol analysis. Univariate and multivariate analyses will be used to compare the outcomes of the intervention group and control group. Regardless of whether intervention patients received one, two, or three interventions, the overall change in 1-year DHI-score in the intervention group will be compared to the change in 1-year DHI-score in the control group. We will use a repeated-measures mixed-effect model to assess differences in the overall change in 1-year DHI score in the intervention group and control group, adjusted for potential confounders. Furthermore subgroup analysis will be performed for three groups separately that received one of three interventions. The overall change in 1-year DHI score in these three subgroups will be compared to the overall change in 1-year DHI score in the control group.

Validation study

Descriptive statistics will be used to describe the study population. Dropout and loss to follow-up will be described. The validity of our seven-item risk score in this study group will be quantified by assessing the reliability, discrimination, and calibration of the model [42]. The reliability of the model will be quantified with the Hosmer-Lemeshow goodness-of-fit statistic. We will

assess the calibration of the model by plotting the predicted probabilities against the observed frequencies of persistent dizziness-related impairment. We will assess the discriminative ability of the model, that is, its ability to distinguish patients with dizziness and persistent dizziness-related impairment from patients with dizziness but without persistent dizziness-related impairment, by calculating the receiver operating characteristic curve (AUC). An AUC of 0.5 indicates no discrimination above chance, whereas an AUC of 1.0 indicates perfect discrimination. We will bootstrap to adjust for over-optimism in model performance.

Discussion

This study is expected to add evidence on the effectiveness of a prognosis-oriented approach for dizziness in older people in primary care. The risk factor-guided intervention of this prognosis-oriented approach will provide the GP with more tools for treatment, even if diagnosis is not (yet) available. An important aim of the intervention study is to reduce dizziness-related impairment in older patients. The intervention may also lead to a decrease in healthcare costs by a reduction of healthcare utilisation in secondary and tertiary care. Furthermore, the availability of a new effective intervention for older patients with dizziness may stimulate GPs to reduce unnecessary drug prescribing [43]. Alongside the intervention study we will validate a previously developed seven-item risk score for chronic dizziness with persistent impairment [19]. Once validated, this risk score will enable GPs to identify patients at high risk for chronic dizziness with persistent impairment.

A strength of this study is that it is, to date, the first study that will investigate the effectiveness of a prognosis-oriented approach on reducing dizziness-related impairment in a large group of older people in primary care. Several researchers have suggested that a multi-component intervention targeting contributory factors of the geriatric syndrome dizziness may reduce dizziness and dizziness impairment [4, 8, 15, 17, 18, 21]. Yet this study goes even further by applying a prognosis-oriented approach instead of a general multifactorial approach. The prognosis-oriented approach focuses on modifiable risk factors for an unfavourable course of dizziness, whereas the multifactorial approach focuses on random contributory factors for the geriatric syndrome dizziness. Also, Croft et al. recently argued that patient prognosis can provide the framework for modern clinical practice for more effective and efficient care [14]. Another strength of this study is the pragmatic design, enabling evaluation of the outcomes in real-life routine practice conditions. The pragmatic design increases the generalizability of our outcomes and increases the possibility of implementing the prognosis-oriented approach in daily clinical practice.

Due to the nature of the interventions, it will not be possible to blind patients and caregivers to the interventions. To avoid contamination within practices, we have chosen a cluster randomised design: GPs and MHNPs in control practices will not receive training, as opposed to GPs and MHNPs in intervention practices who will. Offering several interventions to a single patient might make measurement of the effects of the separate interventions impossible. However, based on earlier research we expect that the majority of patients (60 %) will be eligible for only one intervention (additional analysis, [26]). This will also enable us to analyse the effects of the individual interventions.

In summary, the overall aim of this study is to demonstrate that a prognosis-oriented approach is more effective than usual care when treating dizziness in older patients in primary care. We will do this by measuring the effectiveness of a multi-component intervention for dizziness. Alongside the intervention study we will validate a previously developed seven-item risk score for chronic dizziness with persistent impairment.

Trial status

The collection of data started in January 2015. The first study results are expected in 2016.

Abbreviations

AUC: Area under the curve; DHI: Dizziness Handicap Inventory; EMR: Electronic medical record; EQ-5D-5L: Euro Quality of Life-5-dimension, 5-level; FRID: fall-risk-increasing drug; GAD: Generalised anxiety disorder; GAD-7: Generalised Anxiety Disorder-7; GP: General practitioner; MDD: Major depressive disorder; MHNP: Mental health nurse practitioner; PD: Panic disorder; PHQ-9: Patient Health Questionnaire-9; PHQ-PD: Patient Health Questionnaire Panic Module; PST: problem-solving treatment.

Competing interests

The authors declare that they have no competing interests.

Authors' contributions

HS, JvdW, HvdH, and OM designed the study. HS conducts the study and wrote the first draft of this manuscript. JvdW, HvdH, and OM provided important intellectual revision of the manuscript. All authors read and approved the final version of this manuscript.

Acknowledgments

This research project is funded by the Netherlands Organisation for Health Research and Development. The funding body did not and will not have any role in the trial design, collection, analysis, or interpretation of data, nor in the writing the manuscript.

Received: 9 April 2015 Accepted: 2 July 2015
Published online: 25 July 2015

References

1. Maarsingh OR, Dros J, Schellevis FG, Van Weert HC, Bindels PJ, Van der Horst HE. Dizziness reported by elderly patients in family practice: prevalence, incidence, and clinical characteristics. BMC Fam Pract. 2010;11:2.
2. Aggarwal NT, Bennett DA, Bienias JL, de Leon CFM, Morris MC, Evans DA. The prevalence of dizziness and its association with functional disability in a biracial community population. J Gerontol A Biol Sci Med Sci. 2000;55(5):M288–92.
3. Cigolle CT, Langa KM, Kabeto MU, Tian Z, Blaum CS. Geriatric conditions and disability: the health and retirement study. Ann Intern Med. 2007;147(3):156–64.
4. Tinetti ME, Williams CS, Gill TM. Dizziness among older adults: a possible geriatric syndrome. Ann Intern Med. 2000;132(5):337–44.

5. Lasisi AO, Gureje O. Disability and quality of life among community elderly with dizziness: report from the Ibadan study of ageing. J Laryngol Otol. 2010;124(9):957–62.
6. Sloane P, Blazer D, George LK. Dizziness in a community elderly population. J Am Geriatr Soc. 1989;37(2):101–8.
7. Colledge NR, Wilson JA, Macintyre CC, MacLennan WJ. The prevalence and characteristics of dizziness in an elderly community. Age Ageing. 1994;23(2):117–20.
8. Sloane PD, Coeytaux RR, Beck RS, Dallara J. Dizziness: state of the science. Ann Intern Med. 2001;134(9 Pt 2):823–32.
9. Dros J, Maarsingh OR, Beem L, Van der Horst HE, Ter Riet G, Schellevis FG, et al. Impact of dizziness on everyday life in older primary care patients: a cross-sectional study. Health Qual Life Outcomes. 2011;9(1):44.
10. Tinetti ME, Williams CS, Gill TM. Health, functional, and psychological outcomes among older persons with chronic dizziness. J Am Geriatr Soc. 2000;48(4):417–21.
11. Hartholt KA, Polinder S, Van der Cammen TJM, Panneman MJM, Van der Velde N, Van Lieshout EMM, et al. Costs of falls in an ageing population: a nationwide study from the Netherlands (2007–2009). Injury. 2012;43(7):1199–203.
12. The National Institute for Health and Care Excellence. Clinical knowledge summaries: vertigo. http://cks.nice.org.uk/vertigo. Accessed 17 March 2015.
13. Verheij AAA, Van Weert HCPM, Lubbers WJ, Van Sluisveld ILL, Saes GAF, Eizenga WH, et al. The guideline "Dizziness" of the Dutch College of General Practitioners. Huisarts Wet. 2002;45:601–9.
14. Croft P, Altman DG, Deeks JJ, Dunn KM, Hay AD, Hemingway H, et al. The science of clinical practice: disease diagnosis or patient prognosis? Evidence about "what is likely to happen" should shape clinical practice. BMC Med. 2015;13:20.
15. Kao AC, Nanda A, Williams CS, Tinetti ME. Validation of dizziness as a possible geriatric syndrome. J Am Geriatr Soc. 2001;49(1):72–5.
16. Inouye SK, Studenski S, Tinetti ME, Kuchel GA. Geriatric syndromes: clinical, research, and policy implications of a core geriatric concept. J Am Geriatr Soc. 2007;55(5):780–91.
17. Gassmann KG, Rupprecht R. Dizziness in an older community dwelling population: a multifactorial syndrome. J Nutr Health Aging. 2009;13(3):278–82.
18. Gomez F, Curcio CL, Duque G. Dizziness as a geriatric condition among rural community-dwelling older adults. J Nutr Health Aging. 2011;15(6):490–7.
19. Dros J, Maarsingh OR, Beem L, Van der Horst HE, Ter Riet G, Schellevis FG, et al. Functional prognosis of dizziness in older adults in primary care: a prospective cohort study. J Am Geriatr Soc. 2012;60(12):2263–9.
20. De Moraes SA, Soares WJ, Ferriolli E, Perracini MR. Prevalence and correlates of dizziness in community-dwelling older people: a cross sectional population based study. BMC Geriatr. 2013;13:4.
21. Maarsingh OR, Stam H, Van de Ven PM, Van Schoor NM, Ridd MJ, Van der Wouden JC. Predictors of dizziness in older persons: a 10-year prospective cohort study in the community. BMC Geriatr. 2014;14(1):133.
22. Maarsingh OR, Stam H, Van der Horst HE. A different approach of dizziness in older patients: away from the diagnostic dance between patient and physician. Front Med (Lausanne). 2014;1:50.
23. Campbell MK, Piaggio G, Elbourne DR, Altman DG. Consort 2010 statement: extension to cluster randomised trials. BMJ. 2012;345:e5661.
24. Jacobson GP, Newman CW. The development of the Dizziness Handicap Inventory. Arch Otolaryngol Head Neck Surg. 1990;116(4):424–7.
25. Tamber A-L, Wilhelmsen KT, Strand LI. Measurement properties of the Dizziness Handicap Inventory by cross-sectional and longitudinal designs. Health Qual Life Outcomes. 2009;7:101.
26. Maarsingh OR, Dros J, Schellevis FG, Van Weert HC, Van der Windt DA, Ter Riet G, et al. Causes of persistent dizziness in elderly patients in primary care. Ann Fam Med. 2010;8(3):196–205.
27. Van der Velde N, Stricker BHC, Pols HA, Van der Cammen TJM. Risk of falls after withdrawal of fall-risk-increasing drugs: a prospective cohort study. Br J Clin Pharmacol. 2007;63(2):232–7.
28. Van der Velde N, Van den Meiracker AH, Pols HA, Stricker BHC, Van der Cammen TJM. Withdrawal of fall-risk-increasing drugs in older persons: effect on tilt-table test outcomes. J Am Geriatr Soc. 2007;55(5):734–9.
29. Balkom ALJM, Van Vliet IM, Emmelkamp PMG, Bockting CLH, Spijker J, Hermens MLM, et al. [Multidisciplinary guideline anxiety disorders (third revision)] (in Dutch). 2013. http://www.ggzrichtlijnen.nl/index.php?pagina=/richtlijn/pagina.php&fSelectNTG_22=6&fSelectedSub=22. Accessed 17 March 2015.
30. Spijker J, Bockting CLH, Meeuwissen JAC, Van Vliet IM, Emmelkamp PMG, Hermens MLM, et al. [Multidisciplinary guideline depression (third revision)] (in Dutch). 2013. http://www.ggzrichtlijnen.nl/index.php?pagina=/richtlijn/item/pagina.php&richtlijn_id=88. Accessed 17 March 2015.
31. Spitzer RL, Kroenke K, Williams JBW, Löwe B. A brief measure for assessing generalized anxiety disorder: the GAD-7. Arch Intern Med. 2006;166(10):1092–7.
32. Spitzer RL, Kroenke K, Williams JB. Validation and utility of a self-report version of PRIME-MD: the PHQ primary care study. Primary Care Evaluation of Mental Disorders. Patient Health Questionnaire. JAMA. 1999;282(18):1737–44.
33. Arroll B, Goodyear-Smith F, Crengle S, Gunn J, Kerse N, Fishman T, et al. Validation of PHQ-2 and PHQ-9 to screen for major depression in the primary care population. Ann Fam Med. 2010;8(4):348–53.
34. Phelan E, Williams B, Meeker K, Bonn K, Frederick J, Logerfo J, et al. A study of the diagnostic accuracy of the PHQ-9 in primary care elderly. BMC Fam Pract. 2010;11:63.
35. Cuijpers P, Muñoz RF, Clarke GN, Lewinsohn PM. Psychoeducational treatment and prevention of depression: the "Coping with Depression" course thirty years later. Clin Psychol Rev. 2009;29(5):449–58.
36. Mynors-Wallis L, Davies I, Gray A, Barbour F, Gath D. A randomised controlled trial and cost analysis of problem-solving treatment for emotional disorders given by community nurses in primary care. Br J Psychiatry. 1997;170(Feb):113–9.
37. Podsiadlo D, Richardson S. The timed "Up & Go": a test of basic functional mobility for frail elderly persons. J Am Geriatr Soc. 1991;39(2):142–8.
38. De Vries NM, Van Ravensberg CD, Hobbelen JSM, Olde Rikkert MGM, Staal JB, Nijhuis-van der Sanden MWG. Effects of physical exercise therapy on mobility, physical functioning, physical activity and quality of life in community-dwelling older adults with impaired mobility, physical disability and/or multi-morbidity: a meta-analysis. Ageing Res Rev. 2012;11(1):136–49.
39. Brown CJ, Flood KL. Mobility limitation in the older patient: a clinical review. JAMA. 2013;310(11):1168–77.
40. Herdman M, Gudex C, Lloyd A, Janssen M, Kind P, Parkin D, et al. Development and preliminary testing of the new five-level version of EQ-5D (EQ-5D-5L). Qual Life Res. 2011;20(10):1727–36.
41. de Vries OJ, Peeters GMEE, Elders PJM, Muller M, Knol DL, Danner SA, et al. Multifactorial intervention to reduce falls in older people at high risk of recurrent falls: a randomized controlled trial. Arch Intern Med. 2010;170(13):1110–7.
42. Moons KGM, Kengne AP, Grobbee DE, Royston P, Vergouwe Y, Altman DG, et al. Risk prediction models: II. External validation, model updating, and impact assessment. Heart. 2012;98(9):691–8.
43. Maarsingh OR, Schellevis FG, Van der Horst HE. Looks vestibular: irrational prescribing of antivertiginous drugs for older dizzy patients in general practice. Br J Gen Prac. 2012;62(603):518–20.

Submit your next manuscript to BioMed Central and take full advantage of:

- **Convenient online submission**
- **Thorough peer review**
- **No space constraints or color figure charges**
- **Immediate publication on acceptance**
- **Inclusion in PubMed, CAS, Scopus and Google Scholar**
- **Research which is freely available for redistribution**

Submit your manuscript at
www.biomedcentral.com/submit

BioMed Central
